# Persistent symptoms are associated with long term effects of COVID-19 among children and young people: Results from a systematic review and meta-analysis of controlled studies

**DOI:** 10.1371/journal.pone.0293600

**Published:** 2023-12-28

**Authors:** Sanaz Behnood, Fiona Newlands, Lauren O’Mahoney, Mahta Haghighat Ghahfarokhi, Mohammed Z. Muhid, Jake Dudley, Terence Stephenson, Shamez N. Ladhani, Sophie Bennett, Russell M. Viner, Rowan Bhopal, Paige Kolasinska, Roz Shafran, Olivia V. Swann, Andrea Takeda

**Affiliations:** 1 Usher Institute of Population Health Sciences and Informatics, The University of Edinburgh, Edinburgh, United Kingdom; 2 UCL Great Ormond Street Institute of Child Health, London, United Kingdom; 3 Diabetes Research Centre, University of Leicester, Leicester, United Kingdom; 4 Kings College London, London, United Kingdom; 5 University College London, London, United Kingdom; 6 Immunisation Department, MRCPCH(UK), UK Health Security Agency, London, United Kingdom; 7 Department of Child Life and Health, University of Edinburgh, Edinburgh, United Kingdom; 8 Centre for Medical Informatics, Usher Institute of Population Health Sciences and Informatics, The University of Edinburgh, Edinburgh, United Kingdom; 9 Freelance Systematic Reviewer, Winchester, United Kingdom; Kyung Hee University School of Medicine, REPUBLIC OF KOREA

## Abstract

**Background:**

Research on the long-term impact on COVID-19 in children and young people (CYP) has been published at pace. We aimed to update and refine an earlier systematic review and meta-analysis to assess the current evidence for Post-COVID-19 Condition in CYP.

**Methods:**

Studies from the previous systematic review were combined with studies from a systematic search from July 2021 to November 2022 (registration PROSPERO CRD42021233153). Eligible studies included CYP aged ≤19 years with confirmed or probable SARS-CoV-2 infection and symptoms persisting at least 12 weeks.

**Findings:**

55 studies (n = 1,139,299 participants) were included. Over two-hundred symptoms were associated with Post COVID-19 Condition. Gastrointestinal problems, headaches, cough and fever were among the most prevalent symptoms with rates of 50.2%, 35.6%, 34.7% and 25.8% respectively. Twenty-one symptoms from 11 studies were suitable for meta-analysis. There were significantly higher pooled estimates of proportions of symptoms for altered / loss of smell or taste, dyspnoea, fatigue, and myalgia in CYP with confirmed SARS-CoV-2 infection. Heterogeneity was high suggesting substantial variation amongst the included studies.

**Conclusions:**

Many CYP continue to experience symptoms after SARS-CoV-2 infection. Efforts to aid early identification and intervention of those most in need is warranted and the consequences of COVID-19 for CYP call for long-term follow-up.

## Introduction

Persistent symptoms following COVID-19 are emerging as an important health issue with a broad spectrum of manifestations in adults and in children and young people (CYP). Such persistent symptoms are termed ‘Post-COVID-19 Condition (PCC)’ or ‘Long Covid’. The questions which the current systematic review seeks to address are:

1. What are the most common symptoms that persist at least 12 weeks after SARS-CoV-2 infection
2. What is the prevalence of symptoms that persist for at least 12 weeks after SARS-CoV-2 infection?
3. Are there differences in proportions between SARS-CoV-2 positive CYP and controls for symptoms that persist at least 12 weeks after SARS-CoV-2 infection?

Prevalence estimates of PCC in CYP are extremely variable across different studies [1] and the term ‘Post-COVID-19 Condition’ or ‘Long Covid’ is used differently by different authors. This is, in part, because many studies were conducted prior to an agreed research or clinical case definitions, which have now been published [2, 3]. The definitions include criteria specifying the duration of persisting symptoms after testing (i.e., at least 12 weeks [2]). There is a need for research to consistently apply this criteria of 12 weeks to compare across studies and to enable meaningful conclusions. This review applies the criteria of 12 weeks post-infection while accepting that the precise terms of ‘PCC’ and ‘Long Covid’ will be used inconsistently across various studies.

While some studies have appropriate control groups, the literature remains largely characterised by uncontrolled studies [1] and therefore results must be approached with caution. Chronic non-specific symptoms such as fatigue and headaches are prevalent amongst CYP without underlying health issues [4] and consequently, comparisons with non-infected controlled groups are essential to avoid the overestimation of PCC [1]. However, such controlled studies are themselves not without limitations and are also becoming almost impossible to conduct given the large proportion of CYP who have been infected with SARS-CoV-2 with estimates of up to 98% of secondary school pupils in the UK reporting antibody levels above the limit of detection in March 2022 [5].

Existing reviews have been published exploring PCC in CYP [1, 6–8]. However, these studies are not without limitations. Lopez-Leon et al. (2022) include studies where the duration of persisting symptoms is less than 12 weeks [6], and Jiang et al (2023) include studies by Pinto Pereira et al (2023) [9] and Stephenson et al (2022) [10] with overlapping participant groups. Zheng et al (2023) [7] conducted a systematic review and meta-analysis including data from over 12,000 participants, however, they omit recent large epidemiological studies such as those of Rao et al (2022) [11] and Taquet et al (2022) [12]. There is a need to include such studies to help interpret the prevalence of PCC. Given that such high proportions of CYP have been infected with COVID-19 [5], it will not be possible to have an uninfected comparison group for meta-analyses in the future and a definitive meta-analysis is needed now to provide a comprehensive overview of all the available information.

One of the first systematic reviews of the existing literature on persistent symptoms after SARS-CoV-2 infection in CYP was published in February 2022 which included studies published before July 2021 [1]. The review captured data from over 23,000 CYP from 22 studies, of which five were controlled. Meta-analysis found that the pooled risk difference in SARS-CoV-2 positive CYP compared to uninfected controls was significantly higher for cognitive difficulties, headache, loss of smell, sore throat and sore eyes [1]. However, as this was early in the understanding of PCC, it included symptoms persisting beyond four weeks (i.e. the review was conducted before a wider acknowledgement that PCC was better conceived as representing problems at 12 weeks or more after SARS-CoV-2 infection (1,3)). Research on PCC in CYP has continued to be published at pace, and there is a need to update this review to capture up to date literature, including large epidemiological studies, whilst considering the criteria outlined in the published definitions (1,3). With this in mind, the aim of this update was to conduct a methodologically robust systematic review, applying a criteria of symptoms persisting for a minimum of 12 weeks post-infection, and to conduct a meta-analysis of the current literature to establish the prevalence of PCC in CYP.

## Materials and methods

This updated systematic review and meta-analysis was performed following the Preferred Reporting Items for Systematic Reviewers and Meta-analysis (PRISMA) guidelines (registration PROSPERO CRD42021233153). The PRISMA checklist is presented in S1 Table.

### Eligibility

Studies meeting the following criteria were included:

1. Population: CYP aged 0–19 years with confirmed evidence of SARS-CoV-2 infection but, to improve generalisability, excluded studies where all participants were admitted to intensive care unit (ICU). We also included studies where there was probable or suspected (as defined by a clinician) SARS-CoV-2 to account for those studies published before diagnostics tests were readily available. For studies which included specialised populations, for example immunocompromised children, we extracted the data but did not include them in any quantitative syntheses. If studies had mixed populations and only a subset of participants ≤ 19 years, we included the data for the relevant age-group if these were available in the publication.
2. Study type: any study design excluding systematic reviews, other reviews and case-reports of individual CYP. We included published, preprint and grey literature.
3. Outcomes: the type, prevalence and duration of persistent symptoms and their impact on daily functioning in the study population measured with an average follow-up time ≥ 12 weeks after infection. Where studies reported more than one follow-up time, we extracted data from the longest duration between SARS-CoV-2 infection and reported symptoms.

There were no restrictions or limitations on language, date of acceptance or of publications of studies. Google translate was used to translate any non-English publications.

### Searches

Studies included in our original review (from 1st December 2019 to 31st July 2021) [1] were screened and those reporting follow up at ≥ 12 weeks after infection were retained. An updated systematic search was then conducted by the primary reviewer (SAB) from 31st July 2021 to 2nd November 2022 in seven electronic databases using the same databases and search terms as the original review [1].

### Study selection and data extraction

Seven reviewers (SAB, FN, AT, MHG, LO’M, MZM, JD) independently screened the titles and abstracts of all studies identified by the searches, with two reviewers assessing each record. Disagreements were resolved by a third reviewer.

### Risk of bias

The methodological quality of included studies was assessed independently by SAB, FN, AT, LO’M, MHG, JD, RB, PK and checked by a second reviewer using the Newcastle-Ottawa Scale (NOS) for observational studies [13, 14] and the Joanna Briggs Institute (JBI) critical appraisal checklist for cross-sectional and case-series studies [15, 16].

### Analyses

As in our previous review [1], our primary analysis was restricted to controlled studies: participants with confirmed SARS-CoV-2 infection were compared with subjects who tested negative for SARS-CoV-2 (controls). Meta-analysis was performed for symptoms reported by ≥3 studies. Where symptoms were very similar, these were grouped together (e.g. fatigue and weakness). If a study reported two similar symptoms within one of these groups (e.g. both fatigue and weakness), the symptom with the largest number of respondents was analysed. We used random effects meta-analyses to examine the pooled risk difference in prevalence of each symptom or symptom combination in CYP with confirmed SARS-coV-2 infection compared with controls. Analyses were undertaken using Review Manager 5 (RevMan5) software, version 5.4 with random effects model. Heterogeneity was considered as small if I^2^ (estimate of the proportion of the variance across study estimates due to heterogeneity) was <50%, and large if ≥ 50%. As in the previous review, meta-analysis was only undertaken for symptoms with ≥3 studies providing data.

Our secondary analysis involved calculating pooled prevalence estimates for the prevalence of symptoms reported by CYP: (a) with PCC; (b) who, at some time, had a confirmed SARS-CoV-2 infection. Prevalence estimates were calculated for symptoms reported by at least 5 CYP and similar symptoms were grouped as outlined above. Confidence intervals (CI) were calculated for the pooled prevalence estimates using methods set out by Kirkwood and Sterne [17]. A funnel plot was constructed for meta-analyses containing at least 10 studies in accordance with Cochrane Handbook guidance [18].

## Results

**Fig 1:**
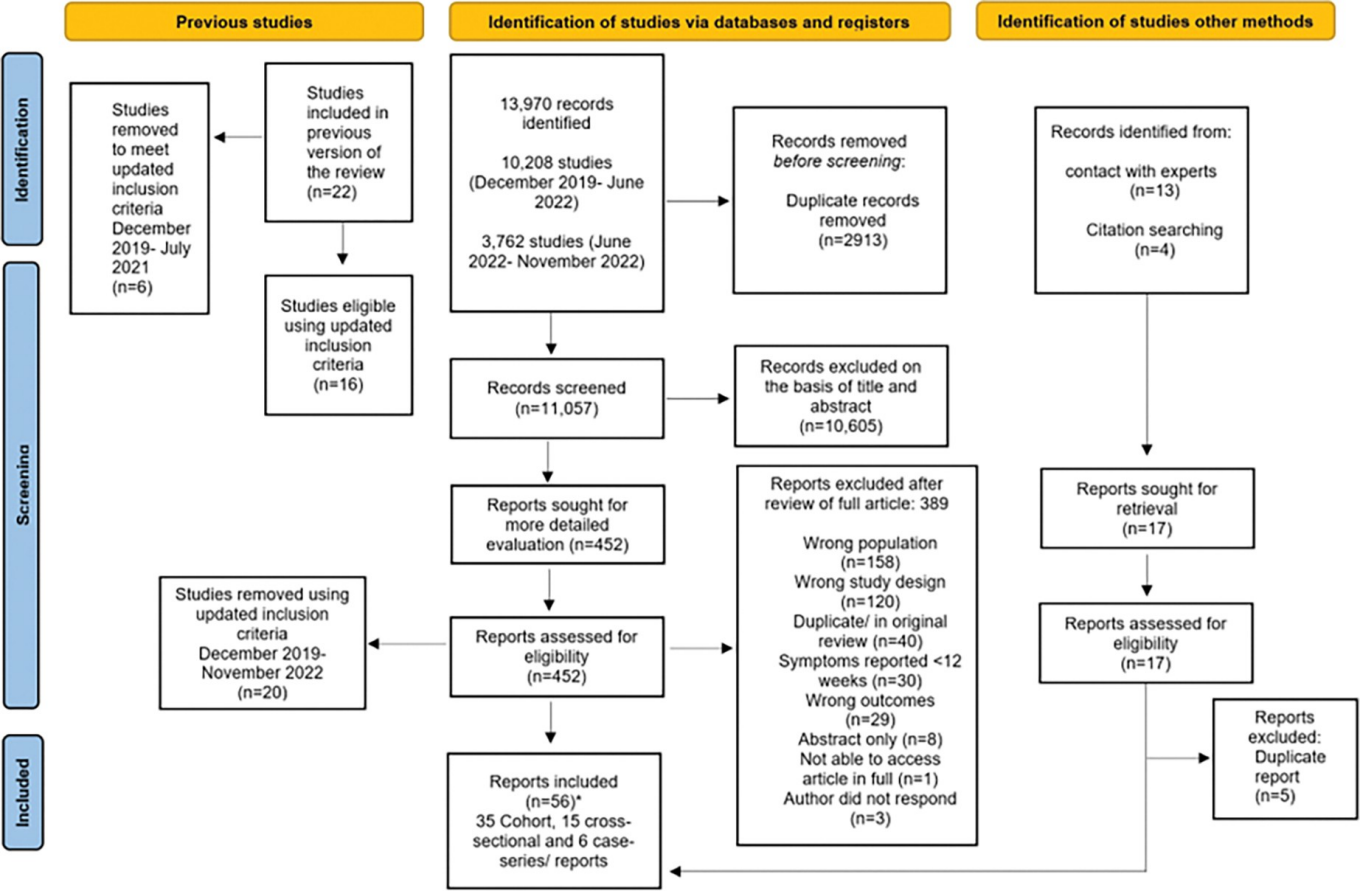
PRISMA flow diagram for included studies [21]. *Two studies used the same methodology and population but report data in separate publications by age group. These have been grouped and analysed together. From [67].

On screening the 22 studies included in our original review [1], 16 reported data on symptoms at or after 12 weeks following infection and so were included in this updated review. In addition, 40 additional studies were identified between July 2021 -November 2022 and are included in the review. Two of the studies used the same population and methodology but presented findings in two publications divided by age group [19, 20]. These two publications were analysed together as one study, leaving a total of 55 studies in this current review. The search flow is shown in Fig 1 with characteristics of the 55 included studies summarised in S2 Table.

Of the 55 included studies, 35 (64%) were cohort studies [10–12, 22–53], 14 (25%) cross-sectional studies [19, 20, 54–66], and 6 (11%) were case reports or series [67–72]. 20 of the 55 studies included population-based control groups [10, 11, 19, 20, 23, 24, 26, 29, 33, 35, 36, 38, 39, 46, 47, 53, 60–63, 65]. 18 (33%) recruited from a mix of previously hospitalised and non-hospitalised [11, 23, 26, 31, 32, 35, 36, 40, 41, 48, 49, 51, 53, 58, 61, 65, 67, 71], 27 (49%) recruited from non-hospitalised CYP [10, 12, 19, 20, 24, 27–29, 33, 37, 38, 45–47, 52, 54–57, 59, 60, 62, 63, 66, 68–70, 72] and 10 (18%) recruited hospitalised CYP [22, 25, 30, 34, 39, 42–44, 50, 64]. Details of studies including hospitalised patients have the proportion admitted to ICU are included in S2 Table.

Sample size ranged from 3 to 659,286 CYP with a total of 1,139,299 participants (median = 148). 23 studies included less than 100 participants [22, 25, 30–32, 43, 45, 48–50, 52, 53, 56, 57, 62, 64–66, 68–72]. All studies assessed outcomes at ≥12 weeks after infection with a range of 87.49 days to over 13 months.

Twenty two studies (40%) were assessed to have high risk of bias [22, 23, 25, 27, 30–32, 34, 39–45, 48–52, 58, 66], eight (15%) moderate [24, 26, 37, 53, 57, 62, 65, 67] and 25 (45%) low risk of bias [10–12, 19, 20, 28, 29, 33, 35, 36, 38, 46, 47, 54–56, 59–61, 63, 64, 68–72]. Two hundred and nineteen symptoms were reported across the 55 studies. Studies included participants from a range of countries including Australia, Canada, Czech Republic, Denmark, Faroe Islands, France, Germany, Israel, Italy, Latvia, the Netherlands, Norway, Poland, Saudi Arabia, Singapore, Spain, Sweden, Switzerland, United Kingdom, and the United States.

### Pooled prevalence estimates

Pooled prevalence estimates for CYP reported to have PCC (as the term was used and defined by the authors of the 62 papers) ranged from 6.6% (loss of appetite; 95% CI 5.2% - 8.1%) to 50.2% (gastrointestinal problems; 49.3%- 51.0%). Headaches, cough and fever were also amongst the most prevalent symptoms for CYP with PCC reported by 35.6% (34.8%- 36.2%), 34.7% (33.9%- 35.5%) and 25.8% (25.1%- 26.5%) respectively. Amongst SARS-CoV-2 infected CYP, prevalence estimates ranged from 1.2% (95% CI 1.1%- 1.3%; cognitive difficulties) to 8.3% (8.0%- 8.5%; fatigue). Pooled prevalence estimates for the remaining symptoms are reported in S4 and S5 Tables.

### Controlled studies

Eleven of the 55 studies included in the review were controlled studies which provided sufficient comparison data and hence were included in the meta-analysis [10, 19, 20, 23, 24, 29, 33, 36, 38, 46, 47, 60]. Two additional studies used control groups but presented findings as hazard ratios, therefore precluding meta-analysis [11, 12]. These studies included 292,978 CYP from Spain, Germany, Italy, Switzerland, Germany and England. One study included data from emergency departments across 8 countries including Argentina, Costa Rica, Paraguay, Singapore and USA. Nine of the studies were assessed to have low risk of bias [10, 19, 20, 29, 33, 36, 38, 46, 47, 60], one moderate [24] and one high [23]. Further characteristics of the 11 studies included in the meta-analysis are summarised in Table 1

**Table 1:** Characteristics of studies included in the meta-analysis.

Study ID (author)	Country	Sample size (n)	Study Design	Age (years) mean*±*SD median (IQR) or [Range]	Sex (% Female)	Baseline severity of COVID-19	Diagnostic Criteria	Duration of Follow-up: mean*±*SD, median (IQR) or [Range]
Bergia [23]	Spain	451 Seropositive 98 Control group	Cohort	Seropositive 4.0 years (IQR 1.0–10.5) Seronegative 7.8 years (IQR 4.1–10.3)	Seropositive 45% Control 43%	82% had mild COVID‐19, 5.1% required PICU admission	PCR, and antigen test or serology	351 days (IQR 330–471 days)
Blankenburg [24]	Germany	188 Seropositive 1365 Seronegative	Cohort	Seropositive: 15 (14–17) Range [10–35] Seronegative: 15 (14–16) Range [10–38]	55% Seropositive 56% Seronegative	NR	Serology (100%)	>3 months
Clavenna [29]	Italy	148 children (41 +ve, 107 –ve)	Cohort	+ve: 7 (4–11.5) -ve: 6 (3–10)	+ve 54% -ve 49%	9.8% hospitalized, 1 later to ICU: length of hospital stays: 5 days to 1 month;	RT-PCR or serology	6 months
Dumont [60]	Switzerland	570 Seropositive 464 Seronegative	Cross-sectional	9.3 (SD 4.5)	49.4%	NR	Tested for anti-SARS-CoV-2 antibodies	>12 weeks
Donnachie [33]	Germany	Cases 43,903 73,873 Controls	Cohort	Range [0–17]	NR	NR	RT-PCR (100%)	24 months after diagnosis of COVID-19
Funk [36]	Includes emergency department data from 8 countries (Argentina, Canada, Costa Rica, Italy, Paraguay, Singapore, Spain and USA)	1884 cases 1701 controls	Prospective cohort	Median 3 (IQR 0–10)	47.2%	Severe acute illness in 18.6% of hospitalised children	Nucleic acid test	90 days after emergency department visit
Haddad [38]	Germany	544 (140 adolescents, 404 children < 14 years old) Infected: 334 Exposed: 210	Part of a prospective observational cohort study	16 (1) for adolescents, 8 (4) for children	49.8%	NR	positive RT- PCR or seropositive on at least 2/3 commercial antibody tests	11–12 months
Kikkenborg Berg, 2022a & b [19,20]	Denmark	CYP: 10997 Cases 33016 Controls Adolescents: 6630 Cases 21640 Controls	Cross Sectional	CYP Cases: 10.2 (6.6–12.8) Control: 10.6 (6.9–12.9) Adolescents: 17.6 (16.4–18.5)	CYP 48% Adolescents:58%	CYP: 54% asymptomatic 44% mild, 2% severe Adolescents: 34% asymptomatic 57% mild, 9% severe	RT-PCR (100%)	>12 months after diagnosis of COVID-19*
Radtke [46]	Switzerland	Seropositive 109 Seronegative 1246	Cohort	Range [6–16]	53% seropositive 54% seronegative	None hospitalised	Serology (100%)	>4 weeks, >12 weeks and 6-month follow-up after serological testing
Roessler [47]	Germany	Cases 11950 Control 59750	Cohort	Range [0–17]	48.1% Cases	98.6% outpatient, 1% hospitalised, 0.4% in ICU	100% Laboratory confirmed diagnosis of COVID-19	≥3 months after COVID-19 diagnosis
Stephenson [10]	England	3065 RT-PCR + 3739 RT-PCR -	Cohort (Preprint)	Age: 11–15 PCR + (56%) Age: 16–17 PCR + (44%) Age: 11–15 PCR—(57%) Age: 16–17 PCR—(43%)	64% PCR + 63% PCR -	65% of PCR + asymptomatic 35% of PCR + symptomatic 92% of PCR—asymptomatic 8% of PCR- symptomatic	RT-PCR (100%)	14.9 weeks (13.1–18.9) after testing

### Meta-analysis

**Fig 2:**
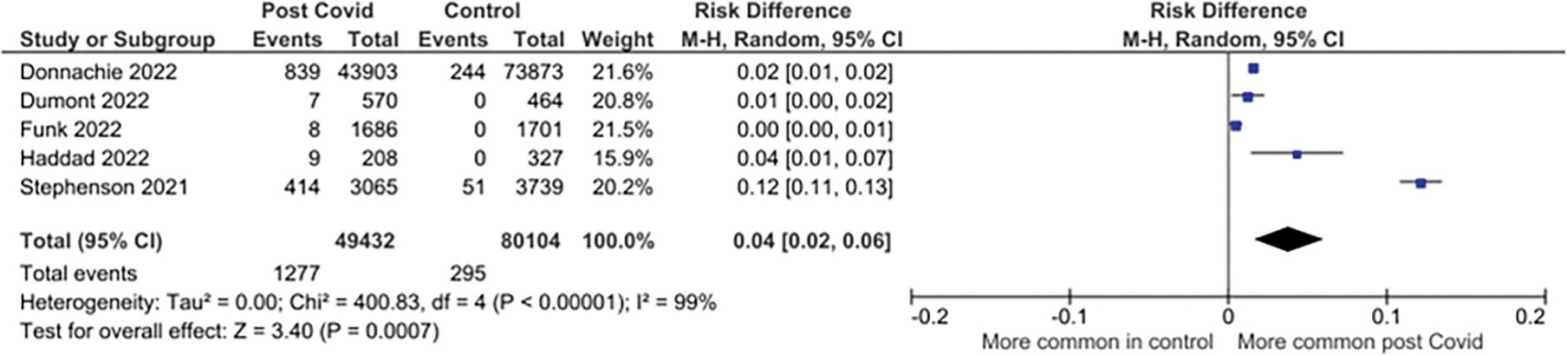
Forest plot of risk difference in symptom prevalence between cases and control participants in controlled studies: Altered/loss of smell or taste.

**Fig 3:**
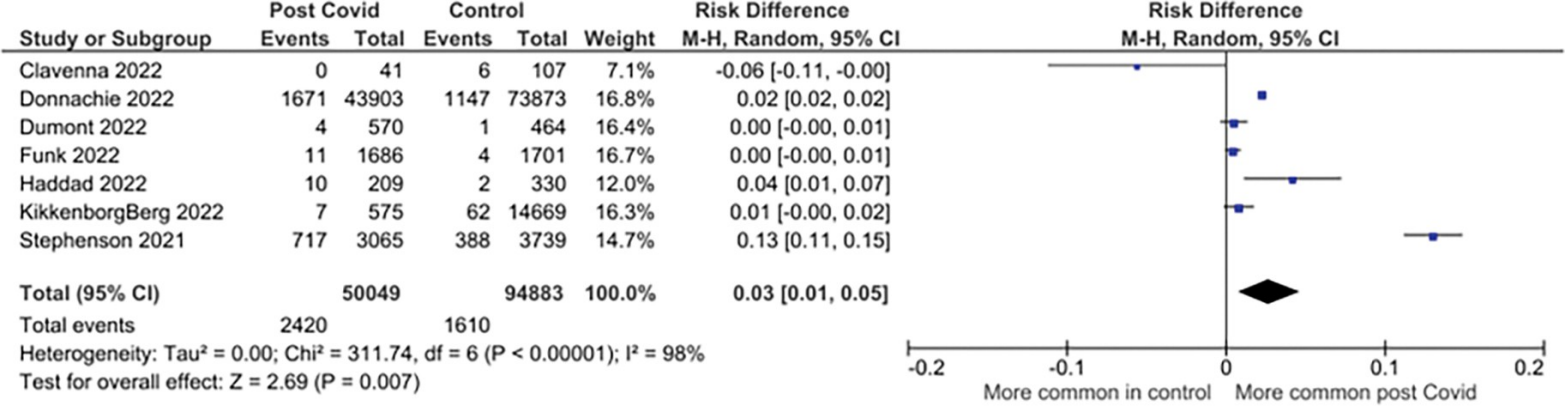
Forest plot of risk difference in symptom prevalence between cases and control participants in controlled studies: Dyspnoea.

**Fig 4:**
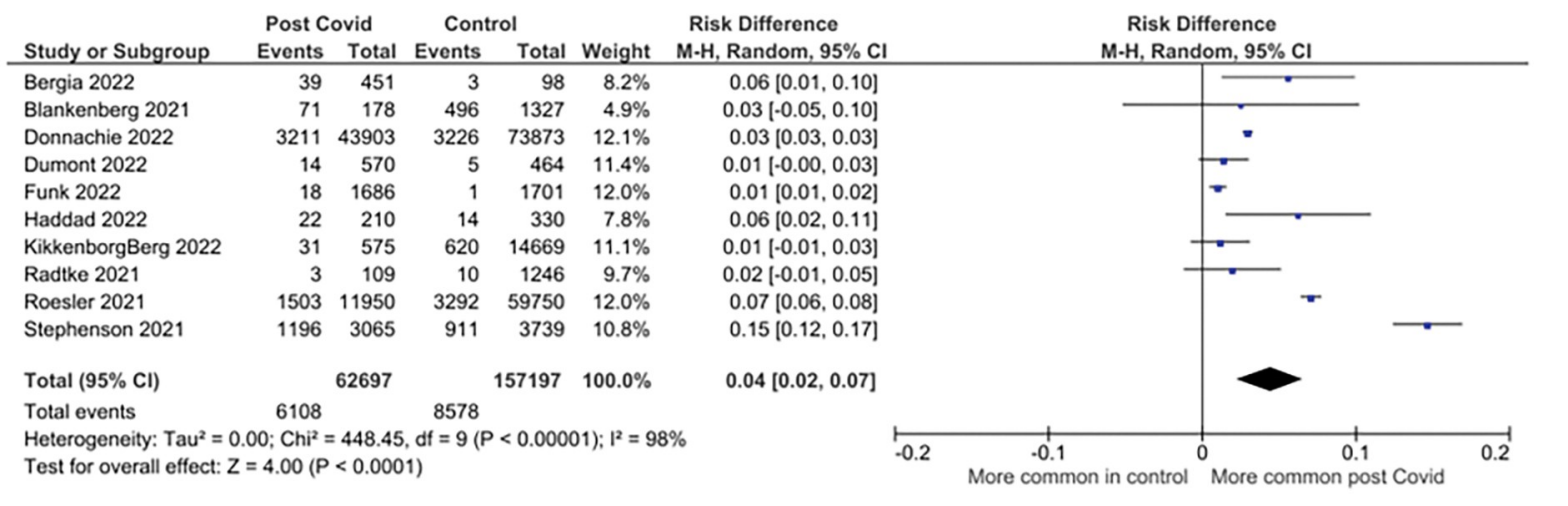
Forest plot of risk difference in symptom prevalence between cases and control participants in controlled studies: Fatigue.

**Fig 5:**
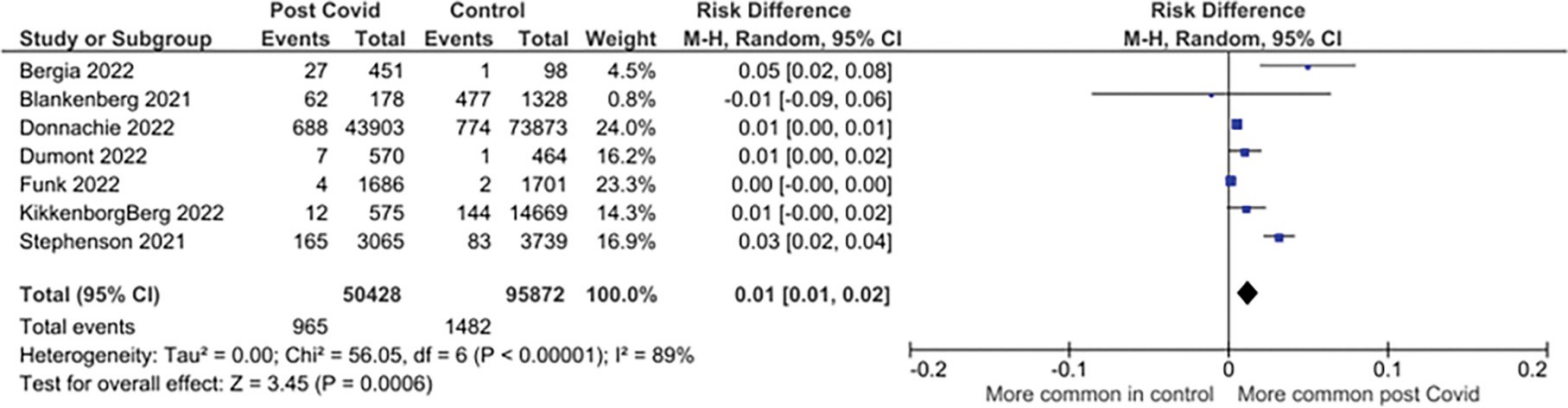
Forest plot of risk difference in symptom prevalence between cases and control participants in controlled studies: Myalgia.

Eleven studies reporting on 68 symptoms provided sufficient data for inclusion in the meta-analysis. Twenty-one symptoms were suitable for meta-analysis. There were significantly higher pooled estimates of proportions of symptoms in CYP with confirmed SARS-CoV-2 infection for altered / loss of smell or taste (pooled risk difference 4% (95% CI 2% to 6%; I^2^ = 99%)), dyspnoea (3% (1% to 5%; I^2^ = 98%)), fatigue (4% (2% to 7%; I^2^ = 98%)) and myalgia (1% (1% to 2%; I^2^ = 89%)). Heterogeneity was high for all these significant associations. No significant difference in proportions between SARS-CoV-2 positive CYP and controls was seen for other symptoms (Table 2). Twenty-three less frequently reported symptoms were not suitable for meta-analysis as data was only available from <3 studies (symptoms listed in S3 Table). Risk differences are shown in Table 2 and forest plots for the symptoms with significantly higher pooled prevalence estimates between cases and controls in Figs 2–5. Forest plots for the remaining symptoms are in S1 Fig.

**Table 2:** Meta-analyses of risk difference in symptom prevalence between cases and control participants in controlled studies: analyses including symptoms reported in ≥3 studies.

Symptom	Symptoms included	Studies	Participants	Effect estimate RD (95% CI)
**Fatigue**	Fatigue / tiredness / weakness / listlessness / chronic fatigue syndrome	10	219,894	0.04 [0.02, 0.07]
**Myalgia**	Myalgia	7	146,300	0.01 [0.01, 0.02]
**Fever**	Fever	5	98,169	0.02 [-0.05, 0.09]
**Cognitive difficulties**	Cognitive difficulties / brain fog / memory impairment / impaired attention / impaired concentration	9	148,188	0.01 [0.00, 0.02]
**Anxiety**	Anxiety	5	77205	0.02 [-0.05, 0.08]
**Depression**	Depression / sadness/ low mood	4	76670	0.02 [-0.03, 0.06]
**Loss of appetite**	Loss of appetite / skipping meals	5	27018	0.01 [-0.00, 0.03]
**Sleep difficulty**	Insomnia / sleep difficulty / hypersomnia	5	4977	0.00 [-0.01, 0.01]
**Headache**	Headache	8	101577	0.04 [-0.06, 0.14]
**Dyspnoea**	Dyspnoea / wheeze / “respiratory symptoms”	7	144932	0.03 [0.01, 0.05]
**Cough**	Cough	6	99524	0.03 [-0.07, 0.12]
**Chest pain**	Chest pain / chest tightness	6	98707	0.02 [-0.04, 0.09]
**Cardiovascular symptoms**	“Cardiovascular symptoms” / palpitations	4	20214	0.00 [0.00, 0.01]
**Dizziness**	Dizziness / dizziness on standing	5	27018	0.01 [-0.03, 0.05]
**Gastrointestinal symptoms**	Stomach ache / diarrhoea / vomiting / nausea / “gastrointestinal symptoms”	8	101578	0.03 [-0.06, 0.13]
**Rash**	Rash	3	19665	0.00 [0.00, 0.01]
**Other dermatological symptoms**	Dermatological symptoms / blisters / skin peeling / itching skin	3	7986	0.00 [-0.01, 0.01]
**Altered/loss of smell or taste**	Altered smell / altered taste / dysgeusia / anosmia/ageusia / parosmia / loss of smell/ loss of taste	5	129536	0.04 [0.02, 0.06]
**Nasal congestion or rhinorrhoea**	Nasal congestion / rhinorrhoea	3	5776	0.00 [-0.01, 0.01]
**Sore throat**	Sore throat	4	94782	0.03 [-0.03, 0.09]
**Ophthalmologic symptoms**	“Ophthalmologic and / or otolaryngologic symptoms”	4	82440	0.01 [-0.07, 0.09]

Fatigue was the only symptom that included 10 or more studies in the meta-analysis. A funnel plot was constructed, however, due to high heterogeneity (I^2^ = 98%) it is difficult to interpret any asymmetry in the funnel plot as being indicative of publication bias (see S2 Fig)

## Discussion

In this comprehensive update of our systematic review and meta-analysis we identified over two hundred symptoms associated with PCC, across cardiovascular, respiratory, gastrointestinal, musculoskeletal, skin and nervous systems as well as general somatic symptoms. As expected, headaches, cough and fever were among the most prevalent symptoms for CYP with PCC with rates ranging from 25–35% beyond 12 weeks post-infection. Other reviews investigating the characteristics of prolonged and persistent clinical features, at least 3 months post-infection have demonstrated a similar constellation of persistent multisystemic symptoms occurring among CYP [7, 8].

The wide range of symptoms highlights the difficulties in defining, characterising, monitoring and comprehensively managing this complex syndrome which is one of the reasons that we accepted authors’ use of the term PCC rather than imposing a published research or clinical definition. The meta-analysis included only 11 controlled studies with 21 symptoms and found that significantly higher pooled estimates of proportions of symptoms in CYP with confirmed SARS-CoV-2 infection only for altered / loss of smell or taste, dyspnoea, fatigue, and myalgia. Altered/loss of smell or taste was the only symptom with a significantly higher pooled estimate in alignment with our previous meta-analysis [1] and this updated review. This suggests our understanding of the symptoms most associated with PCC is in its early stages, and additional research focusing on persisting symptoms experienced by CYP is required.

The high level of the other symptoms in the controls adds to the challenges of understanding and treating PCC. An important additional consideration for treatment is that the impact of symptoms on daily function and symptoms time-course was rarely reported, making it hard to assess the effect of symptoms on the lives of CYP and whether the CYP in the research studies are the same as those seeking treatment. Furthermore, the existing research and clinical definitions of PCC [2, 3] require symptoms to impact on functioning, so it is only possible to state the prevalence of persisting symptoms and not the portion of CYP who fulfil the definitions of PCC. The lack of long-term follow-up also means that longer term monitoring is needed for CYP who continue to experience symptoms 3 months after initial infection.

The strongest studies included an uninfected, SARS-CoV-2 control group but it is unlikely that there will be additional controlled studies with such a group in future given as they become an increasingly small and select population due to high levels of infection in the general population [5]. Instead, it is likely that research designs will begin to examine the course of PCC and symptom profiles within participants rather than across them.

The majority of the included studies in the review were uncontrolled, retrospective, of poor-to-moderate quality and open to selection bias. Twenty-five studies (45%) had low risk of bias. Furthermore, most of them were from high income countries, limiting generalisability for low- and middle-income countries. The research definition of PCC itself is unlikely to be applicable to low- and middle-income countries where there is a lack of funding, testing is relatively uncommon, and is certainly not future-proof given the de-emphasis on testing globally.

In addition, there is the possibility that the “uninfected, SARS-CoV-2 control groups” includes CYP that are contaminated with CYP who have had previous infections but were not tested or did not seroconvert [73]. Few studies in the review reported symptoms at more than one follow-up point and therefore it is not possible to assess how symptoms may be transitory or intermittent and develop over time. Consequently, such repeated follow-up and assessment is essential to properly understanding PCC. One study with repeated follow-up, the CLoCk study, reported its 12-month findings within non-hospitalised young people aged 11–17 years but was published after the November 2022 search date so could not be included in the current review [9]. This study demonstrated that that the prevalence of many adverse symptoms within participants reported at the time of a positive PCR-test declined over 12-months but also that that adverse symptoms were sometimes reported for the first time at six- and 12-months post-test, particularly tiredness, shortness of breath, poor quality of life, poor well-being and fatigue. In another study from Israel, long term clinical outcomes of SARS-CoV-2 infection were assessed during early (30–180 days) and late (180–360 days) time periods in people aged 0 to over 60 years old [74]. The study demonstrated that patients with mild COVID-19 are at a risk for small number of adverse health outcomes, most of which resolve within a year from their diagnosis and that children had an increased risk of a small number of outcomes within the early time periods, but which then returned to baseline in the late time periods. Similar findings were reported by Hahn and colleagues in a Canadian study of 1,026 CYP aged 8–13 years old over a 76-week period [75]. Authors report the incidence of PCC was 0.4% with most CYP experiencing symptom resolution within 2 weeks of infection. These studies speak to the importance of innovative and detailed longitudinal designs over a prolonged period when investigating the long-term impacts of SARS-CoV-2 infection.

Our findings are subject to a number of limitations. Most meta-analyses had high heterogeneity, almost certainly due to both measurement issues across studies and to differing samples, variable clinical definitions, lack of standard reporting, recruitment strategies and arbitrary follow-up times. Because of this, we used a random effects meta-analysis to take account of unmeasured between-study factors. Our findings were limited by lack of data for many symptoms, particularly combinations of symptoms. Very few studies provided data on the impact of symptoms on daily functioning amongst CYP, evidence of other sequalae of COVID-19, symptoms time-course and the duration of symptoms. Furthermore, we were unable to include two controlled studies (of electronic health records) that met the inclusion criteria but only presented their data as hazard ratios [11, 12]. Although we contacted the authors to see if they had any data that we could add to the meta-analysis, additional information was not available at the present time. Although the paper gives cumulative incidence of symptoms at 2 years in the appendix, it was not possible to include that in the meta-analysis as it would have included data from the < 12 weeks period. To improve the generalisability of findings, studies in which all participants had been admitted to ICU were excluded. Examination of symptoms after ICU admission specifically was not within the scope of our analysis and we are unable to comment on those CYP. Importantly, it was not possible to establish whether symptoms were impairing or not, and that is critical in any estimation of the prevalence of PCC in children and young people. Finally, but importantly, our analyses did not allow for causal attribution. We were, for example unable to estimate what proportion of SARS-CoV-2 positive CYP and controls were already suffering from headaches prior to their SARS-CoV-2 test.

Despite these limitations, the study has a number of strengths. It is the largest and most robust systematic review and meta-analysis to date, using criteria aligned to the WHO and Delphi Consensus definition of PCC in children and young people in terms of symptoms persisting for at least 12 weeks post-infection. The findings suggest that it is important to have control groups to place the findings in a broader context. However, with the absence of negative control groups in future due to the widespread nature of the infection [5], it is going to be important to consider appropriate *comparison* rather than control groups. Understanding the difference between the very high proportion of CYP who meet a definition and have persisting symptoms that are not impairing and those CYP whose symptoms are impairing is critical. Benchmarking the data against the prevalence of symptoms found in populations of CYP pre-pandemic is also important. There are clearly many CYP with persisting symptoms and services are not needed (or able) for them all. Efforts to aid early identification and intervention of those most in need is warranted.

In conclusion, we have provided the most up-to-date systematic review and meta-analysis of persisting symptoms beyond 12 weeks following SARS-CoV-2 infection that has impacted the vast majority of young people across the globe. Given the recency of the pandemic, implications of such infection over a long period of time (years not months) is a health priority.

## Supporting information

S1 Fig Forest plots meta-analyses of risk difference in symptom prevalence between cases and control participants in controlled studies: Analyses including symptoms reported in 3 or more studies. Individual symptoms listed in alphabetical order.
(DOCX)

S2 Fig Funnel Plot for the symptom fatigue. (DOCX)

S1 Table PRISMA checklist. (DOCX)

S2 Table Characteristics of included studies. (DOCX)

S3 Table Symptoms reported in <3 controlled studies and therefore not included in the meta-analysis. (DOCX)

S4 Table Pooled prevalence estimates for symptoms reported by CYP with PCC. (DOCX)

S5 Table Pooled prevalence estimates for symptoms reported by SARS-CoV-2 infected CYP. (DOCX)
