# Supplementary material for: Persistent symptoms are associated with long term effects of COVID-19 among children and young people: Results from a systematic review and meta-analysis of controlled studies
Source: PLoS One. 2023 Dec 28;18(12):e0293600. doi: 10.1371/journal.pone.0293600 (PMC10754445; doi:10.1371/journal.pone.0293600)

# **S1 Figure – Forest plots of risk difference in symptom prevalence between cases and control participants in controlled studies: analyses including symptoms reported in 3 or more studies. Individual symptoms listed in alphabetical order.**

Reported symptom: Altered/loss of smell or taste


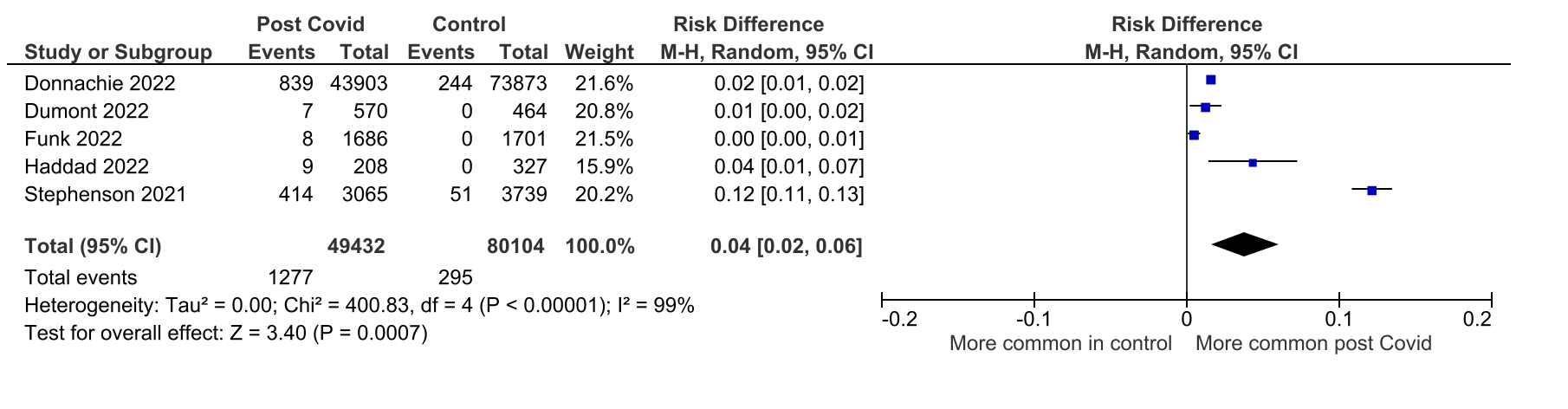


Reported symptom: Anxiety


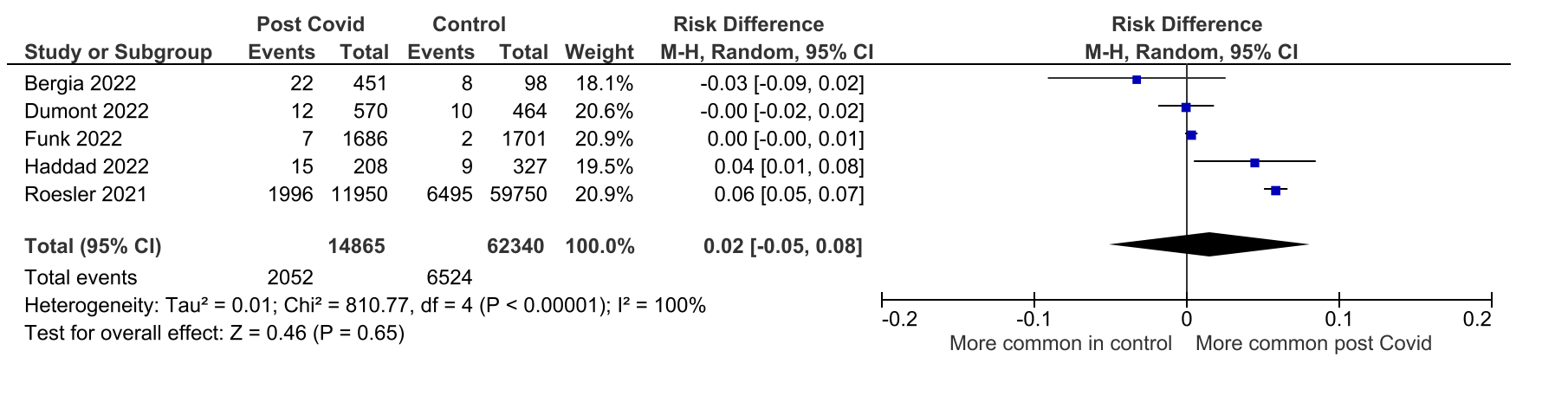


Reported symptom: Cardiovascular symptoms / palpitations


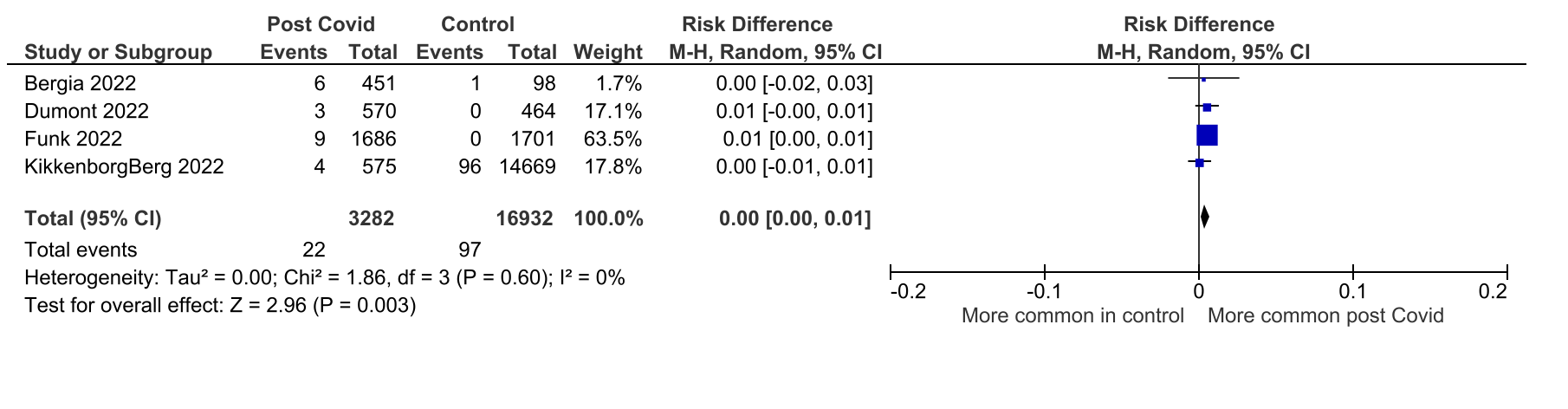


Reported symptom: Chest pain or tightness


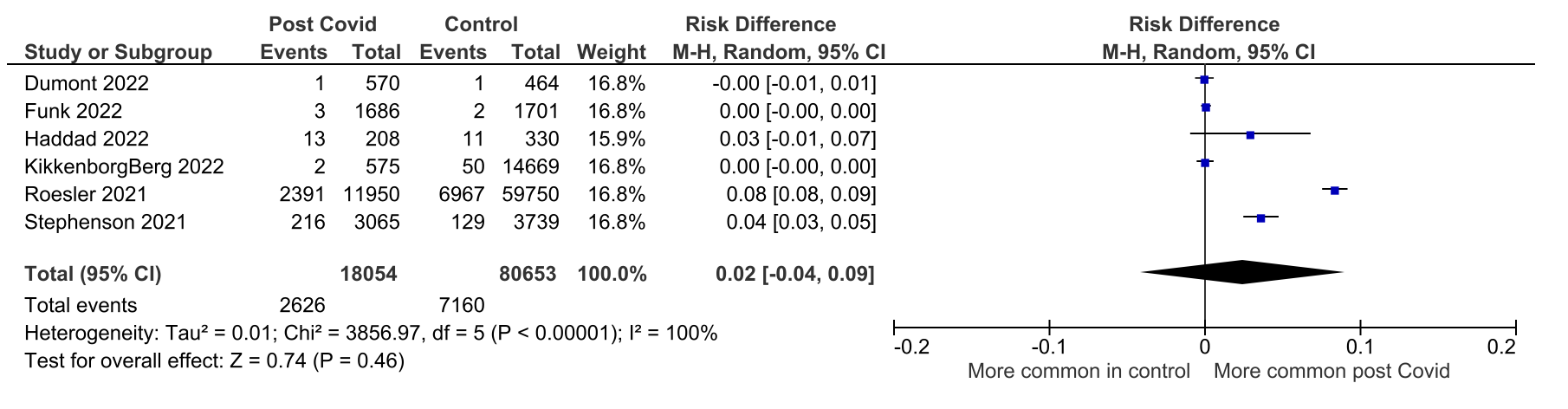


Reported symptom: Cognitive difficulties or brain fog


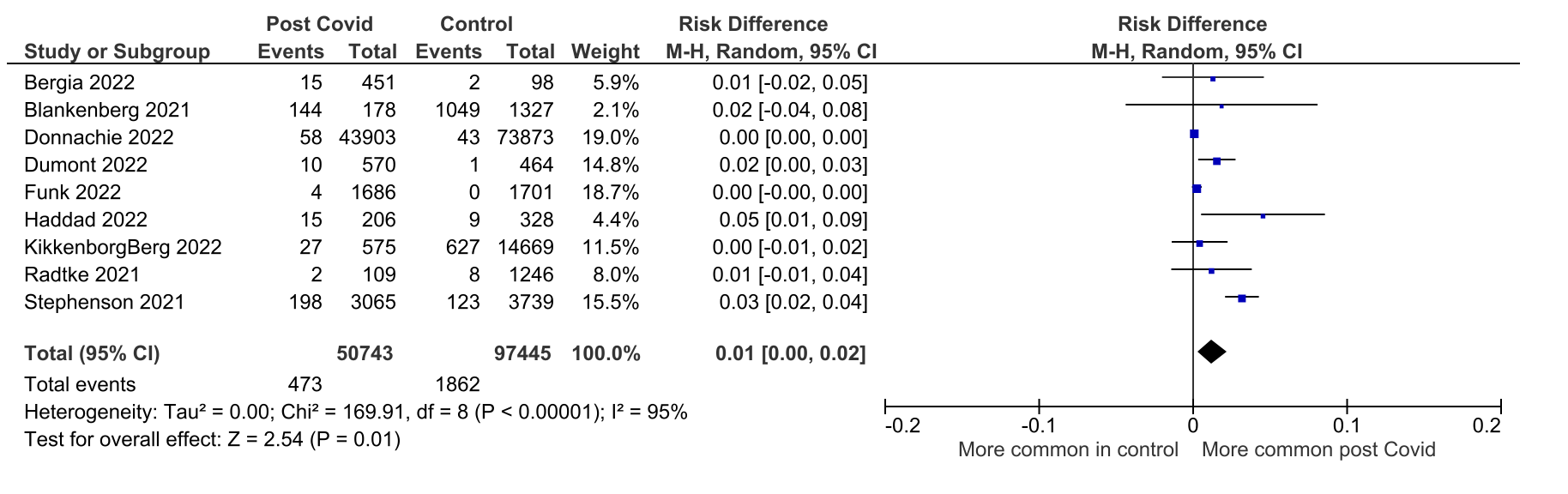


Reported symptom: Cough


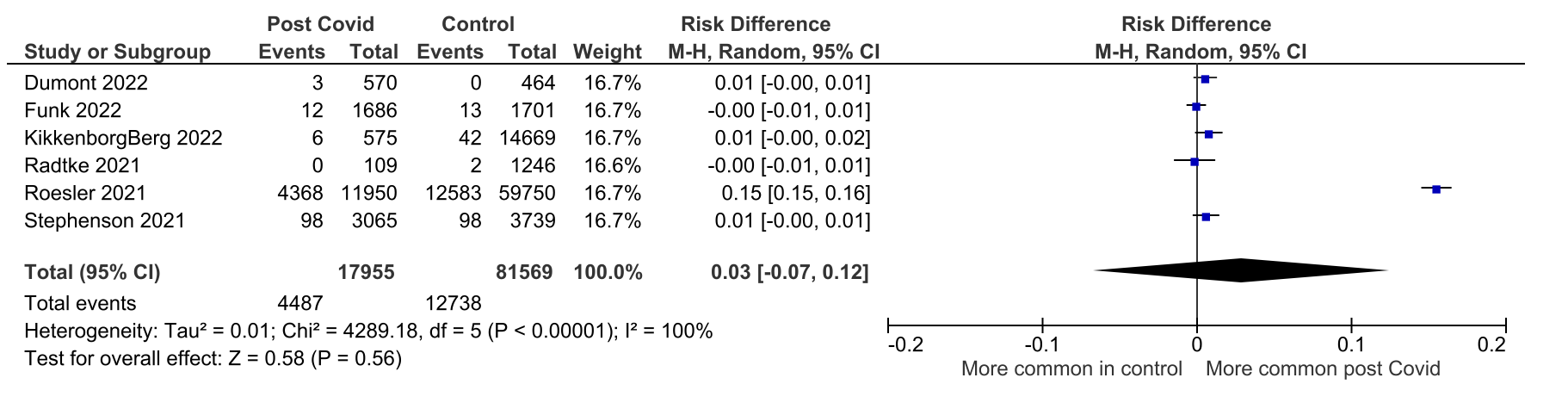


Reported symptom: Depression/ low mood


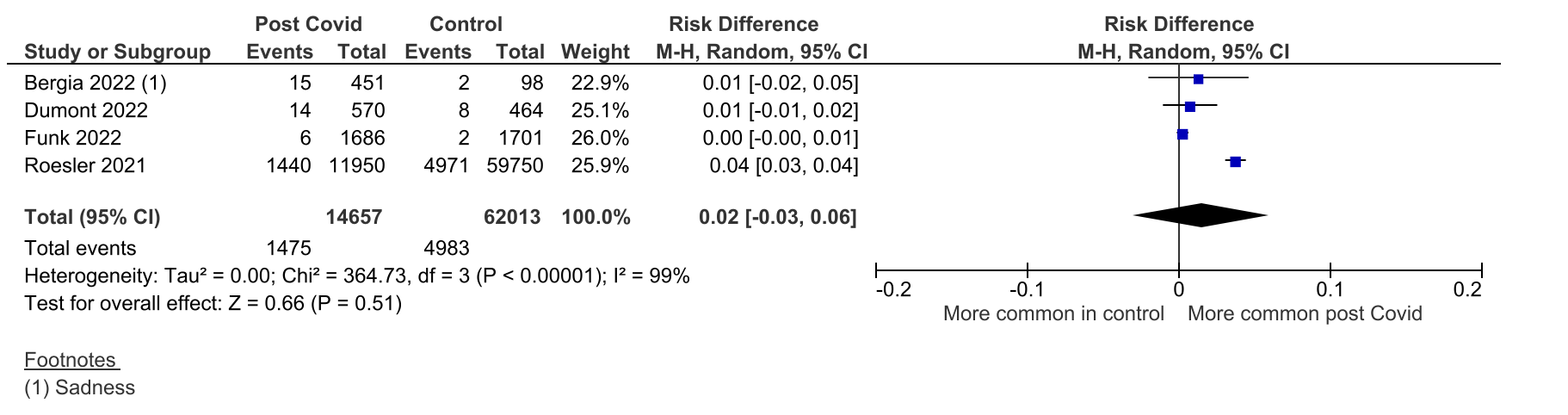


Reported symptom: Dermatological symptoms


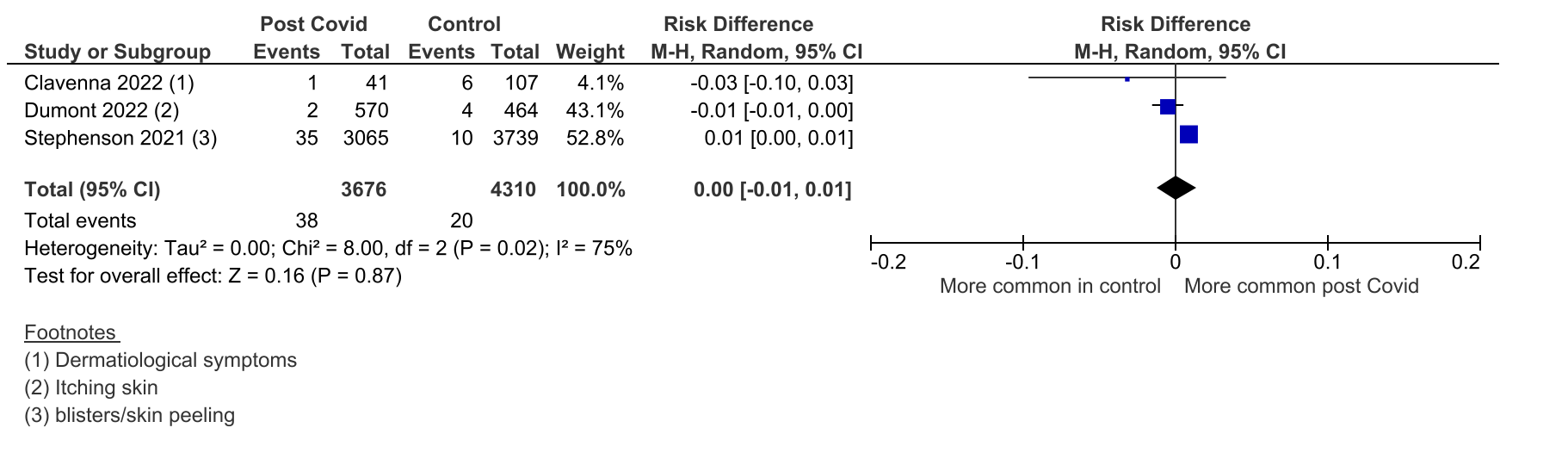


Reported symptom: Dizziness


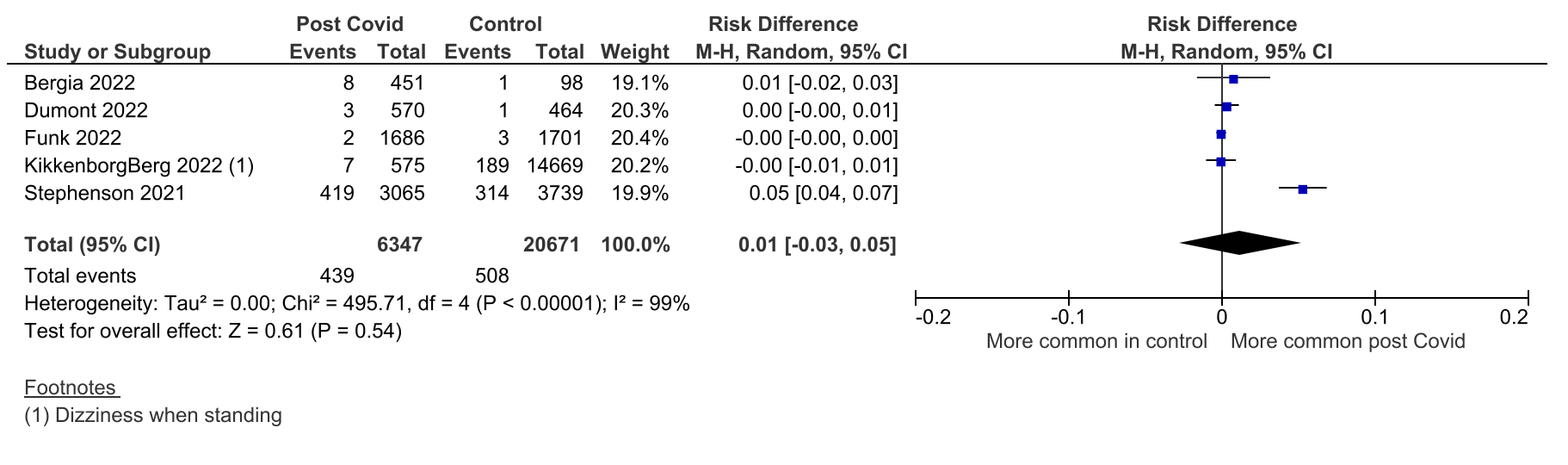


Reported symptom: Dyspnoea or wheezing


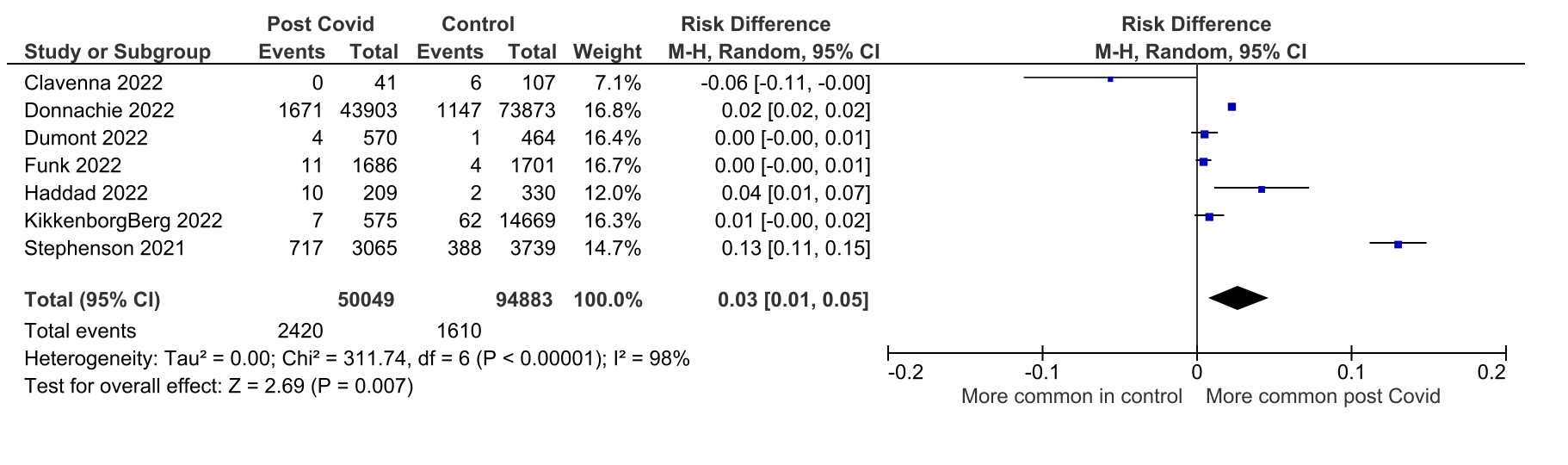


Reported symptom: Fatigue/ weakness


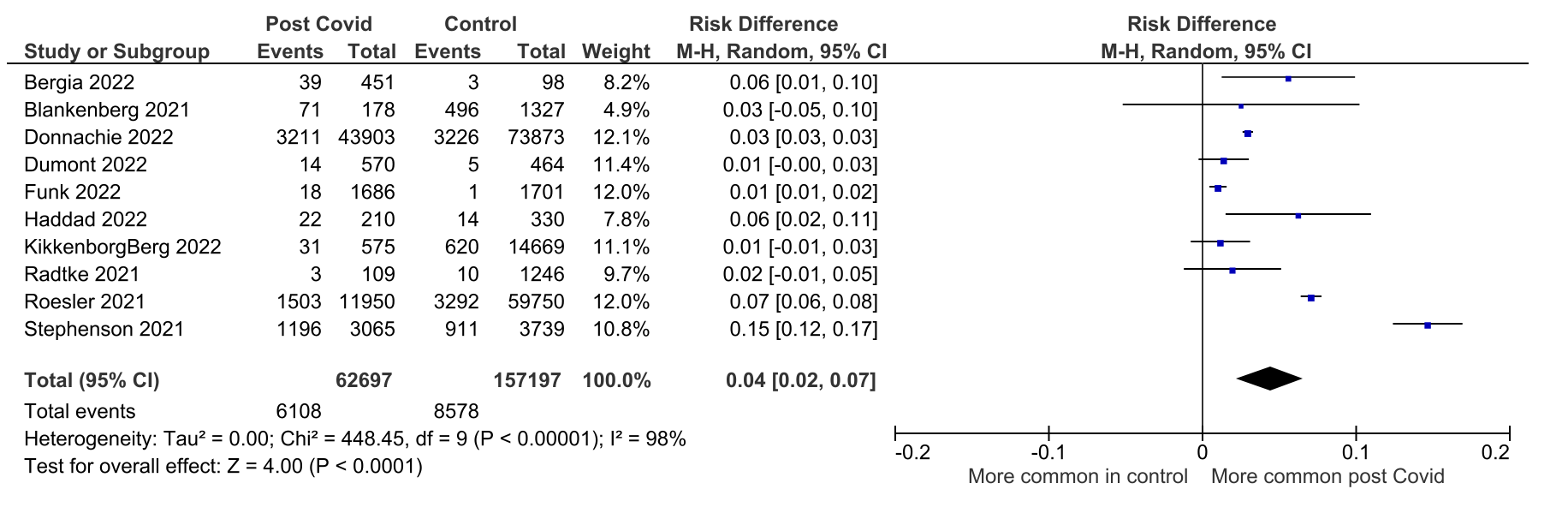


Reported symptom: Fever


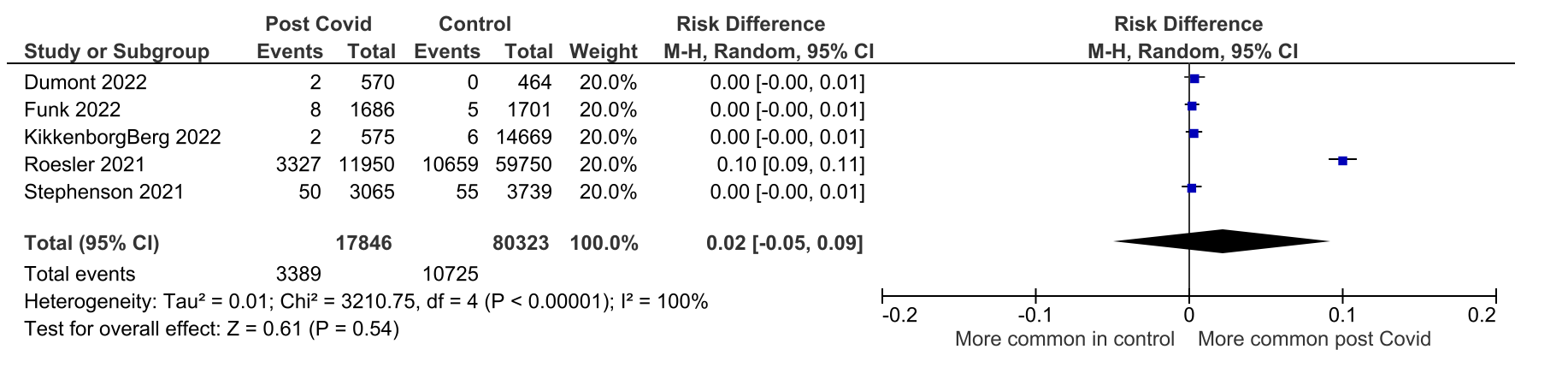


Reported symptom: Headache


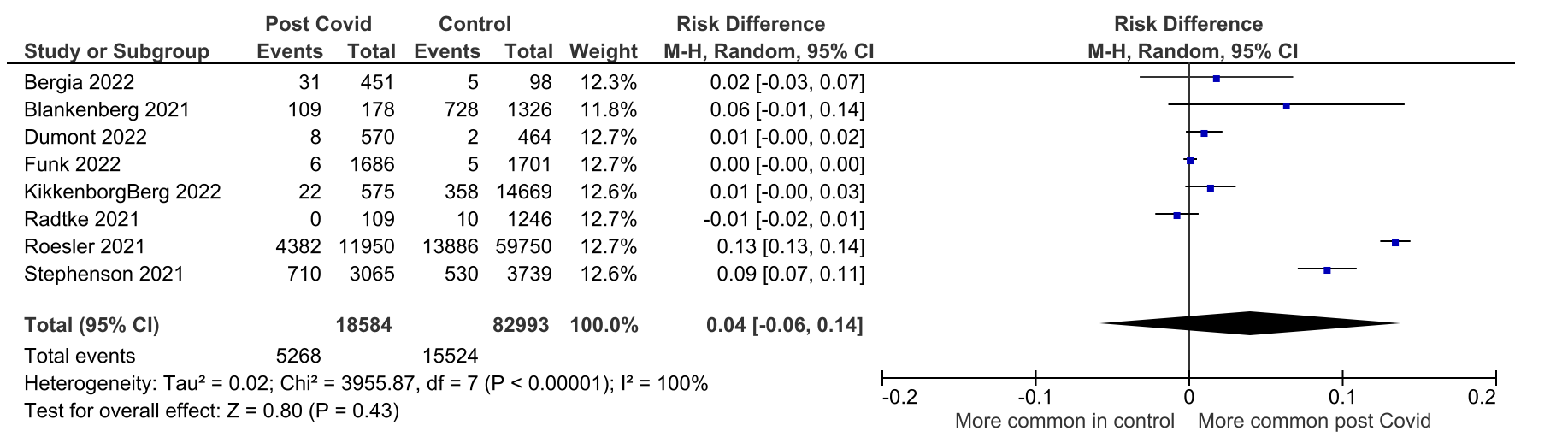


Reported symptom: Insomnia / sleep difficulty


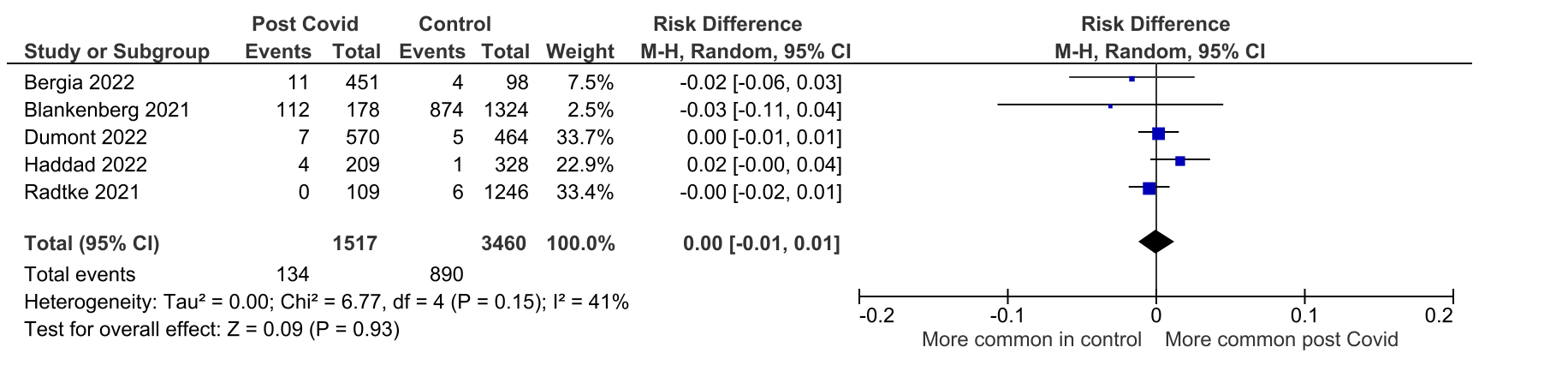


Reported symptom: Loss of appetite


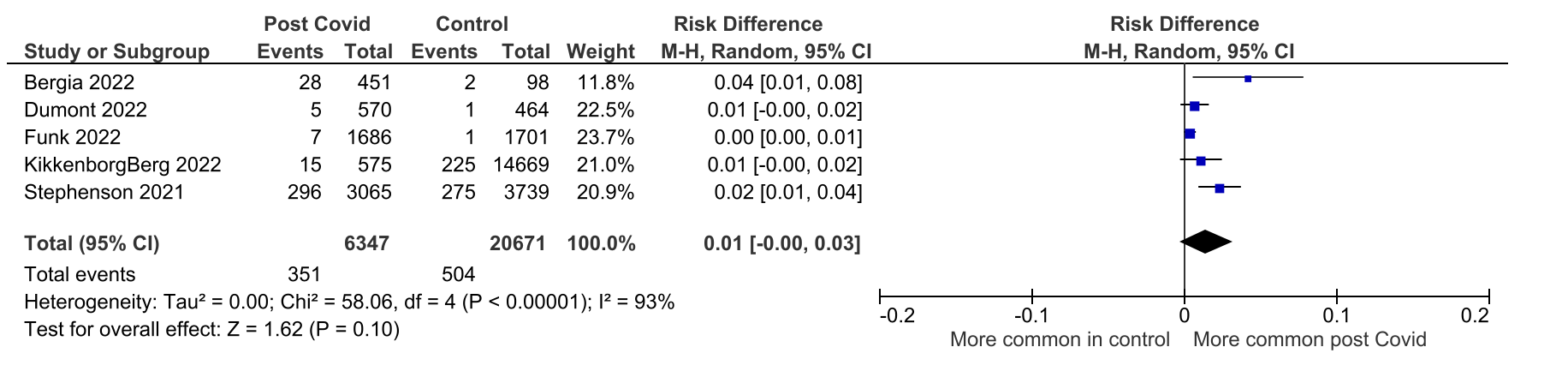


Reported symptom: Myalgia


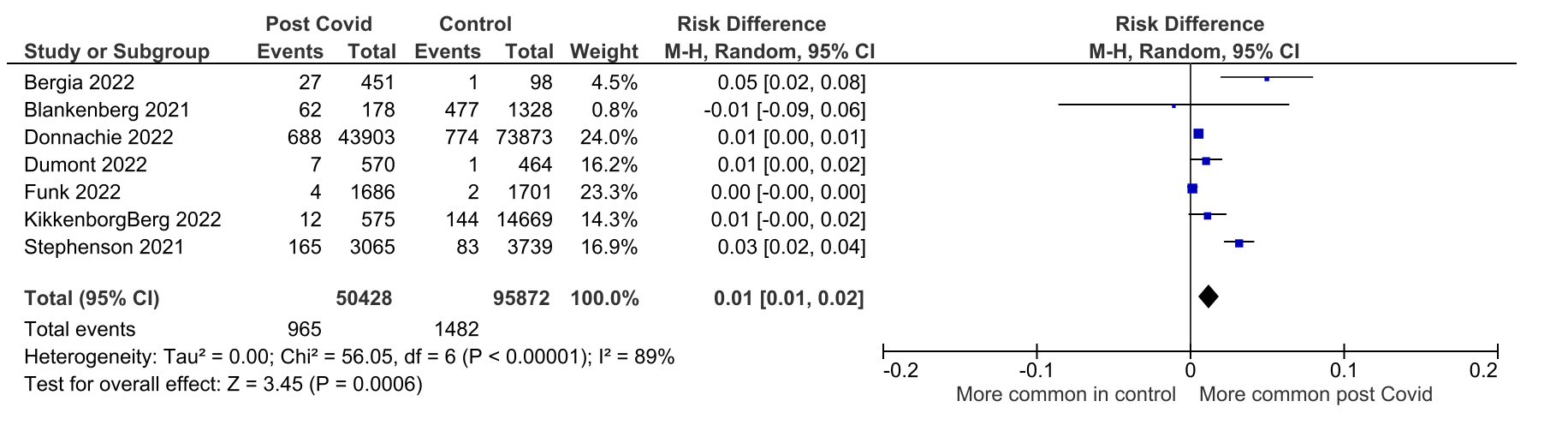


Reported symptom: Nasal congestion or rhinorrhoea


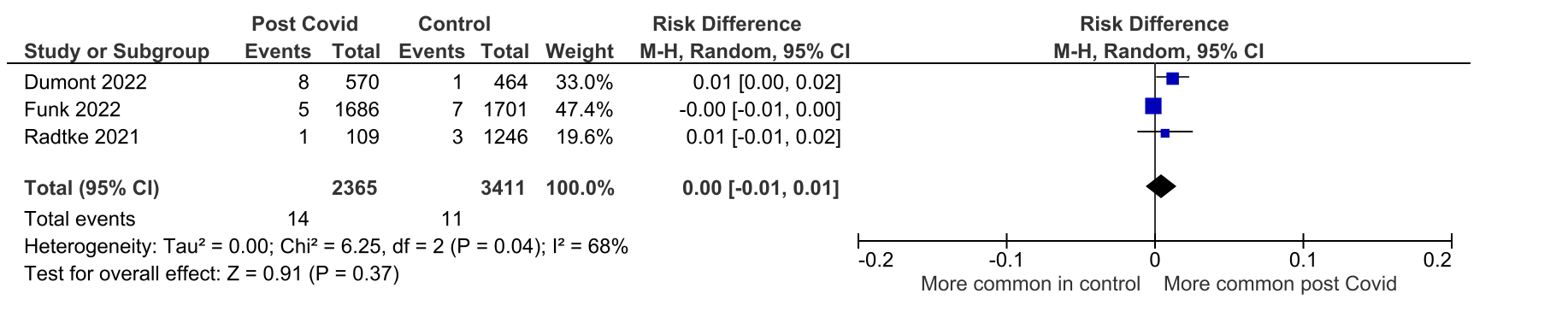


Reported symptom: Ophthalmologic / otolaryngologic symptoms


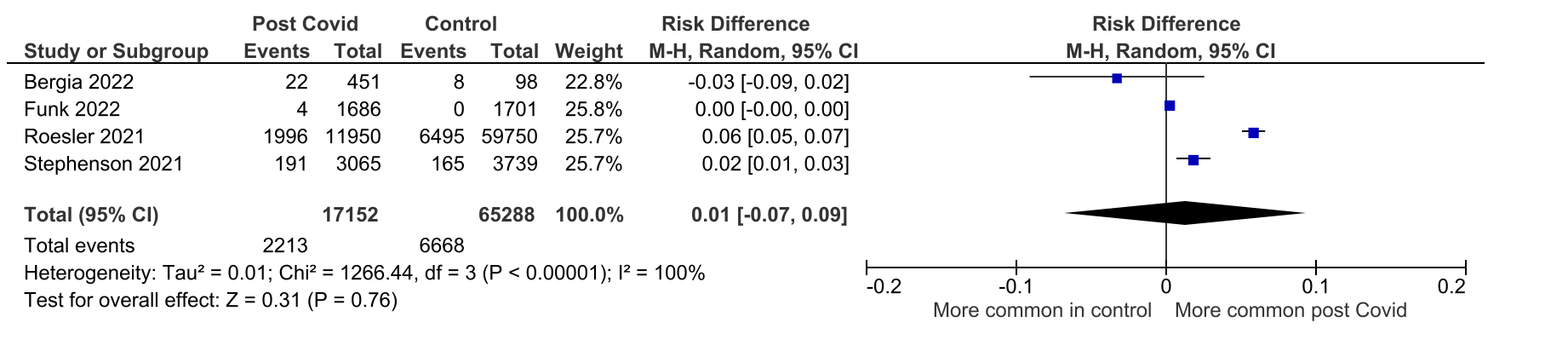


Reported symptom: Rash


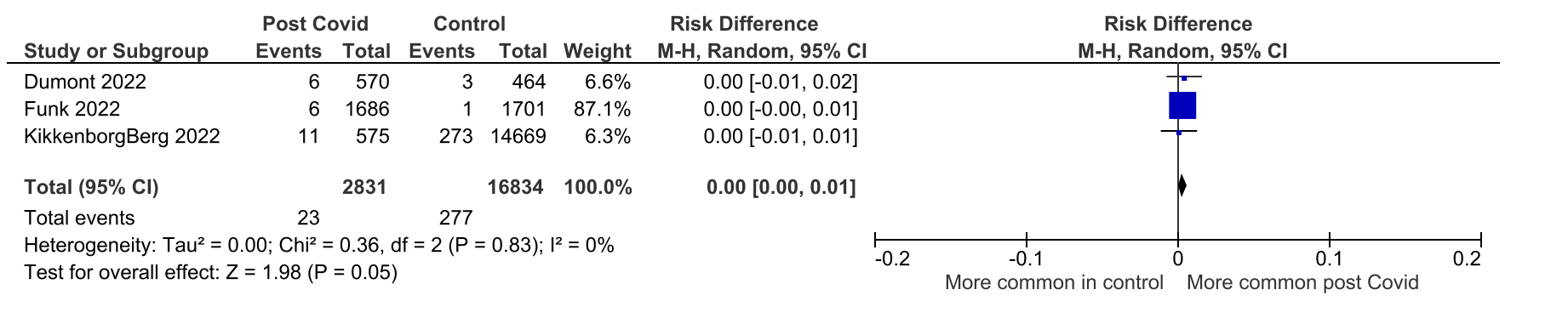


Reported symptom: Sore throat


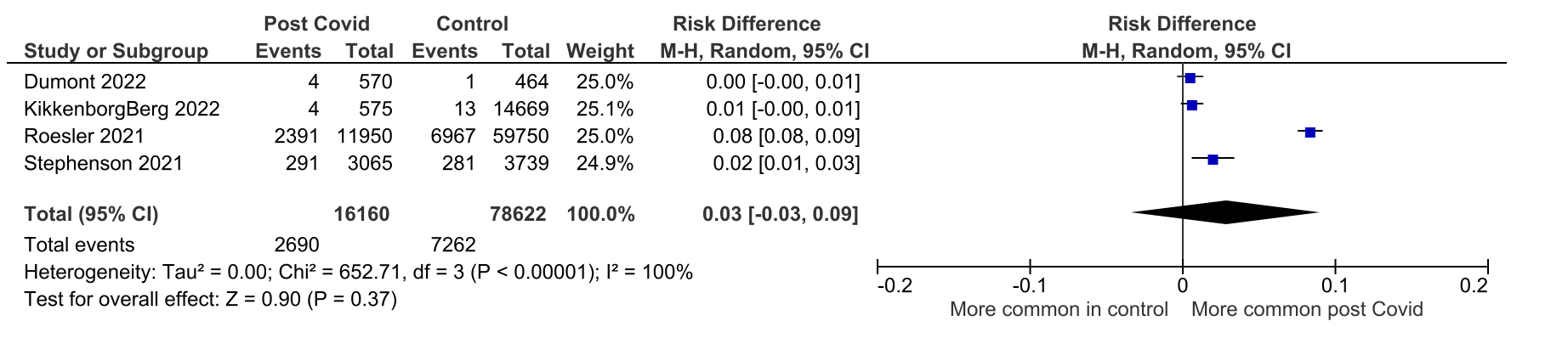


Reported symptom: Stomach ache / gastrointestinal symptoms


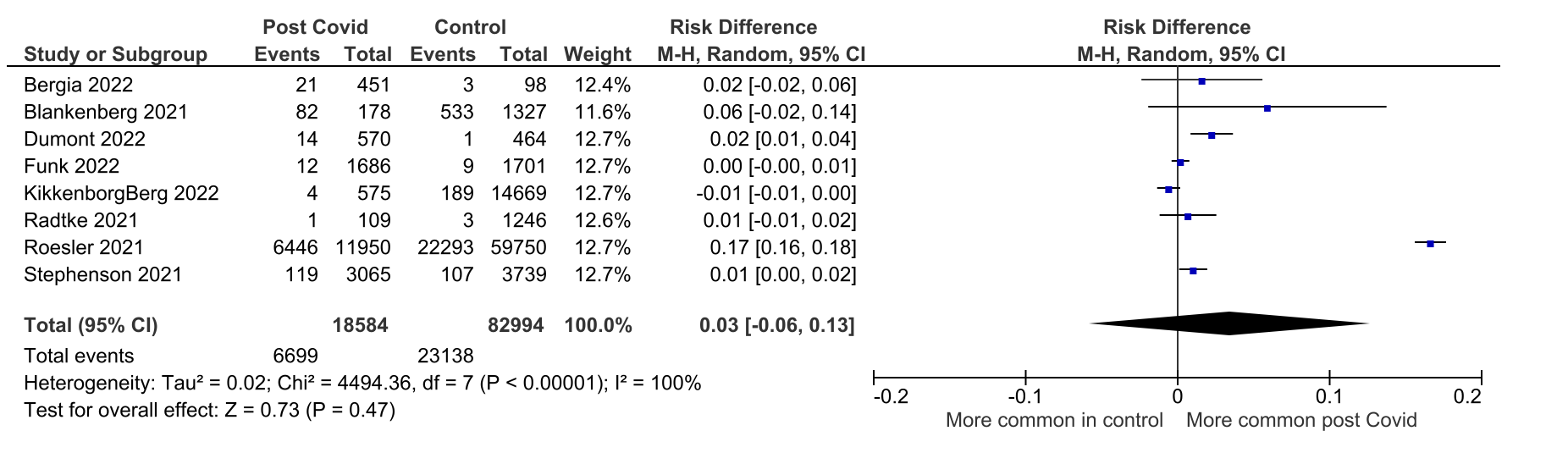

Supplement: S1 Fig — Individual symptoms listed in alphabetical order. (DOCX) [file pone.0293600.s001.docx]
