# Supplementary figures and images for: Persistent symptoms are associated with long term effects of COVID-19 among children and young people: Results from a systematic review and meta-analysis of controlled studies
Source: PLoS One. 2023 Dec 28;18(12):e0293600. doi: 10.1371/journal.pone.0293600 (PMC10754445; doi:10.1371/journal.pone.0293600)

# **Figure 2: Funnel Plot for the symptom fatigue**

**
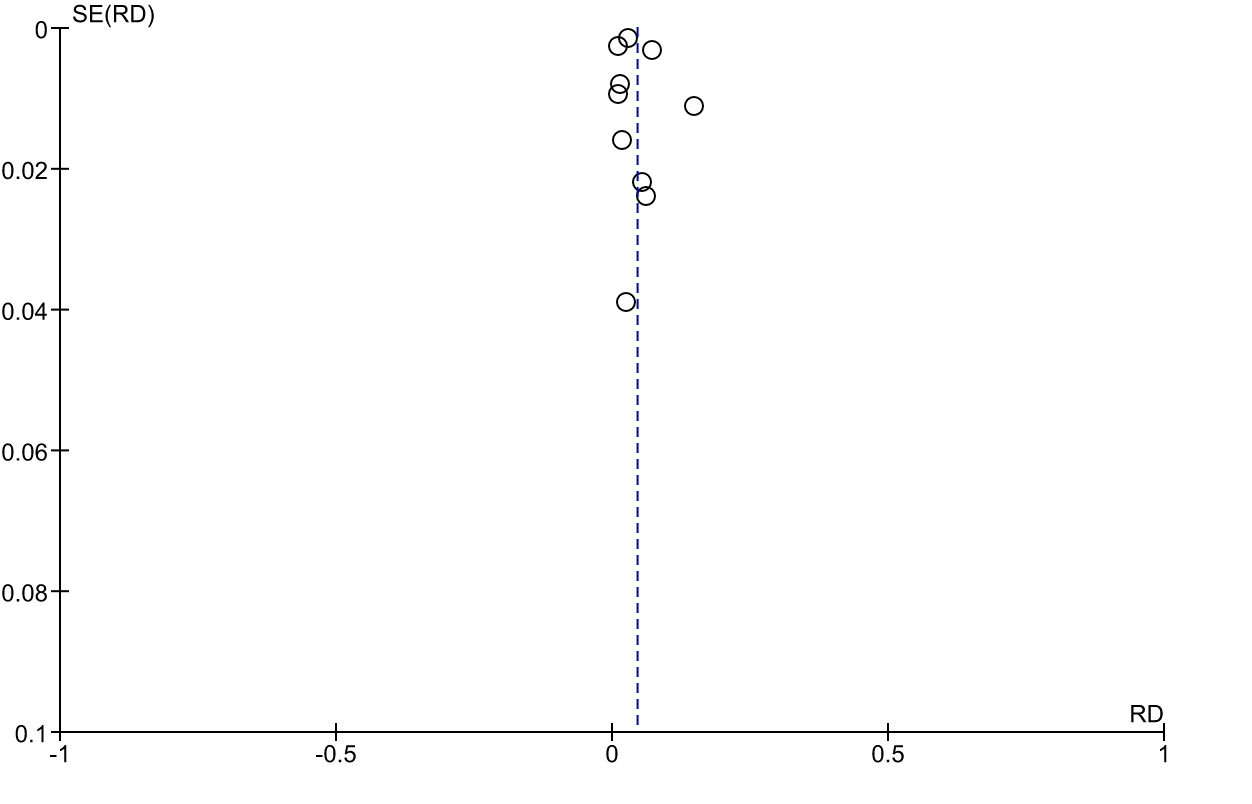
**

Supplement: S2 Fig — (DOCX) [file pone.0293600.s002.docx]
