# Supplementary material for: Persistent symptoms are associated with long term effects of COVID-19 among children and young people: Results from a systematic review and meta-analysis of controlled studies
Source: PLoS One. 2023 Dec 28;18(12):e0293600. doi: 10.1371/journal.pone.0293600 (PMC10754445; doi:10.1371/journal.pone.0293600)
Supplement: S2 Table — (DOCX) [file pone.0293600.s004.docx]

# **S2 Table - Characteristics of included studies**

| **Study ID**   **(author)** | **Country** | **Sample size (n)** | **Study Design** | **Age (years) mean***±***SD**  **median (IQR) or [Range]** | **Sex**  **(% Female)** | **Baseline severity of**   **COVID-19** | **Diagnostic Criteria** | **Duration of Follow-up:**  **mean***±***SD, median (IQR) or [Range]** | **Pre-existing Comorbidities** | **Inclusion Criteria** |
| --- | --- | --- | --- | --- | --- | --- | --- | --- | --- | --- |
| Alghamdi | Saudi Arabia | 153 | Cross-sectional | Range [12-17] | NR by age-group | NR by age-group | RT-PCR (100%) | > 6 months | NR | Positive PCR test, study included 12–17-year-old among other age groups. |
| Asadi-Pooya | Iran | 51 | Cohort study | 13.2 (3.5)years | 55% | 100% initially hospitalised    19.6% needed ICU admission | RT-PCR (100%) | >13 months | 28% had comorbidities  (9% malignancy, 7% diabetes mellitus, 7% asthma) | CYP aged 6-17 years discharged from hospital (alive), having an available phone number registered in the database and oral consent by the parent |
| Bergia | Spain | 451 Seropositive    98  Control group | Cohort | Seropositive 4.0 years (IQR 1.0–10.5)    Seronegative 7.8 years (IQR 4.1–10.3) | Seropositive 45%    Control 43% | 82% had mild COVID‐19,  5.1% required PICU admission | PCR, and antigen test or serology | 351 days (IQR 330–471 days) | Seropositive group    13.3% respiratory chronic diseases or asthma and immunosuppression were the most frequent    Control group- 14.3% | Seropositive group: CYP under 18 yrs. old with diagnosis of SARS‐CoV‐2 infection which had been confirmed by PCR, antigen or serology between 14 March and 31 December  Parents/guardians needed to agree. Excluded CYP with asymptomatic SARS‐CoV‐2 infections. |
| Blankenburg | Germany | 188 Seropositive  1365 Seronegative | Cohort | Seropositive: 15 (14-17)  Range [10-35]  Seronegative:  15 (14-16)  Range [10-38] | 55% Seropositive  56% Seronegative | NR | Serology (100%) | >3 months | NR | 14-17 year-old students in 14 secondary schools with seroprevalence assessment |
| Bloise | Italy | 1413 | Cross Sectional | 10 (IQR: 6 – 13) years | 48.8% | 27.5% asymptomatic, others mild symptoms; 1% hospitalised (3 had pneumonia, 1 had pericarditis with pericardial effusion, 1 had MIS_C). | NR | 87.49 ± 56.44 days after COVID-19 diagnosis | 76.8% had no comorbidities (comorbidities: 2.5% respiratory, 1.6% cardiac, 1.6% gastroenterological, 2.4% neurological, 0.5% nephrological, 1.4% endocrinological, 1% genetic, 0.1% immunodeficiencies, 0.4% rheumatological | Families with children infected by SARS-CoV-2 March 2020-March 2021 living in Latina Local Health Authority. |
| Blomberg | Norway | 33 (16 COVID +ve, 17 seronegative controls) | Cohort study | 8 (IQR 6-12) in COVID +ve group | 56% in COVID +ve group | None in this age group were hospitalised. | RT–PCR and serological antibody positivity. | 6 months | NR by age-group | Home-isolated patients with COVID-19 from 28 February to 4 April 2020, diagnosed with PCR test at Emergency Clinic. Household contacts of infected patients served as secondary cases or seronegative controls. |
| Bode | Germany | 53 | Cross-sectional | Median 9.4  (IQR 3.9) | 47.2% | 100% mild; 32.1% asymptomatic | PCR or serology | 12 months after infection | No pre-existing comorbidities | Children aged < 14 part of the COVID-19 BaWü prospective household study including families with at least one SARS-CoV-2 PCR confirmed or seropositive index case of SARS-CoV-2 were included, between April and August 2020 |
| Bossley | UK | 71 | Cohort | Mean age 6.7  Range [11 days–17 years] | 41% | All hospitalised; of the original cohort (n=88): 27% asymptomatic or incidental findings, 48% mild, 12.5% moderate, 5.5% severe, 7) critical | SARS-CoV-2 RNA positivity | 3-12 months | 49% of original 88 and 5/11 with >4-week symptoms had comorbidities | CYP ≤ 18 years with SARS-CoV-2 RNA positivity, admitted between 1 March 2020 and 19 January 2021 to King’s College Hospital3. None had PIMS-TS |
| Brackel | The Netherlands | 89 | Cross-sectional | 13 (9-15) | NR | 18% hospitalised | RT-PCR - 53%, Serology - 35%,  CD - 38%,  Suspected -  9% | ≥12 weeks after diagnosis of COVID-19 | NR | CYP referred to pediatricians across hospitals in The Netherlands for long-COVID assessment |
| Buonsenso a | UK | 510 | Cross-Sectional (Preprint) | 10.3±3.8 | 56% | 12% asymptomatic74% managed at home  4% hospitalised  9% attended hospital (not admitted) | RT-PCR-28%,  LFT-1%,  CD-31%,   Suspected 41% | >4 weeks after symptom onset^a^ | 56% had comorbidities | CYP with symptoms persisting for more than 4 weeks included. Self-selected from online patient group |
| Buonsenso b | Italy | 129 | Cross-Sectional | 11±4.4 | 48% | 26% asymptomatic74% symptomatic  5% hospitalised,  2% PICU | RT-PCR (100%) | 163 ±114 days after microbiological diagnosis | 10% neurological, 5% skin problems, 4% asthma, 3% allergic rhinitis | All CYP ≤18 years diagnosed with microbiologically confirmed COVID-19 presenting to single hospital |
| Buonsenso c | Italy | SARS-CoV-2 +ve 249      SARS-CoV-2 –ve 37 | Cohort | SARS-CoV-2 +ve 10.4 ± 4.5      SARS-CoV-2 -ve  10.5 ±3.2 | 51% | 2.4% hospitalised and of these 33.3% required PICU | RT-PCR (100%) | Median 77 days (47-169) after COVID-19 diagnosis.^a^ | 52% were reported to have comorbidities and risk factors | CYP ≤18 years old diagnosed with RT-PCR confirmed SARS-CoV-2 infection  between April 1st, 2020 and April 31st, 2021 |
| Buonsenso d | Italy | 169 | Case-series | Range [0-18] | 54% | 13% asymptomatic 79% mild, 7% moderate, 1% severe  7% admitted to hospital and 2% to PICU | NR | 184 days | 9.6% had comorbidities | CYP 0-18 seen at a single post-COVID unit followed at the outpatient setting |
| Castro | USA | 5058 | Cohort  (preprint) | 12.4 (8.9 - 5.6) | 50% | NR | RT-PCR (100%) | 90-150 days | NR | Children aged 5-18 across 2 New England health systems who had a positive SARS-CoV-2 PCR test between 3/12/2020 and 4/18/2021 and at least 90 days of follow-up visits documented in electronic health record. |
| Chevinsky | USA | 305 inpatients   2,368 outpatients | Matched cohort | Range [≤1-17] | 44%  inpatient    51%  outpatient | NR | CD (100%) | Range  31-120 days after diagnosis of COVID-19 | NR | CYP aged <18 years identified from all payer databases including inpatient and outpatient data from April-June 2020 |
| Clavenna | Italy | 148 children (41 +ve,  107 –ve) | Cohort | +ve: 7 (4-11.5)  -ve: 6 (3-10) | +ve 54%  -ve 49% | 9.8% hospitalized, 1 later to ICU:  length of hospital stays: 5 days to 1 month; | RT-PCR or serology | 6 months | 7% cases and 8% controls | children with flu-like symptoms and children with family members who had a suspected or confirmed COVID-19 infection |
| Cocciolillo | Italy | 3 | Case series | 13.3 (range 13-14) | 67% (n=2) | All patients were mild | RT-PCR (100%) | > 3 months | NR | Part of a larger observational study of children < 18 assessed in the outpatient paedetric post-COVID centre after confirmed diagnosis of SARS-CoV-2 infection |
| Denina | Italy | 25 | Cohort | 7.8  Range [0.4-15] | 52% | 28% mild, 56% moderate, 16% severe | Serology or RT-PCR | 130 days from discharge (IQR 106–148) | 1 cystic fibrosis  1 congenital heart disease | CYP admitted to the paediatric COVID-department, with COVID-19 from March 1 to June 1, 2020 |
| Dobkin | USA | 29 | Cohort | 13.1±3.9  Range [4-19] | 59% | 93% symptomatic, 14% hospitalised, 3% MIS-C | RT-PCR or confirmed close household contacts with positive SARS-CoV-2 testing | 3.2 ± 1.5 months  Range [1.3-6.7 months] after SARS-CoV-2 PCR testing or confirmed close household contact | 62% overweight  / Obese,  38% asthma | CYP referred to pulmonary clinic at single hospital with history of SARS-CoV-2 RNA positivity or confirmed close household contact |
| Dolezalova | Czech Republic | 39 (34 for 6-month follow-up) | Cohort | 13.5 (8-15) | 56.4% | Mild; 1 required hospitalisation but no ventilatory support or oxygen therapy; none had MIS-C. | PCR (64.1%), serology 10.3%, both 25.6% | 6 months follow-up for symptom resolution; specific symptoms for > 12 weeks (inclusion criteria). | NR | Paediatric referrals aged 2-18 with persistent respiratory symptoms > 12 weeks after COVID-19. |
| Doshi | USA | 372 | Cohort | 11.10±5.82  Range [0-17] | 52.7% | Severe; 23.7% had 15 or more health care visits over 5-month follow-up. 20.4% were readmitted and 18.0% had an emergency room visit in the 5-months after index hospitalisation. | NR (primary diagnosis of COVID-19) | 5 months | NR | CYP aged 0-17 requiring continuous insurance enrollment 5-months preadmission and 5-months post-admission from hospital with a primary diagnosis of COVID-19, identified by administrative claims data available from Optum Labs. |
| Dumont | Switzerland | Cases 570  Controls 464 | Cross-sectional | 9.3 (SD 4.5) | 49.4% | NR | Tested for anti-SARS-CoV-2 antibodies | 4- 12 weeks +^a^ | NR | Household survey, selected at random |
| Erol | Turkey | Cases  121    Control  95 | Cross Sectional | Cases  9.16 (10.9-17.9)    Control  8.42 (8.7-17.4) | Cases  46%    Control  47% | 9% asymptomatic | “Children who had COVID-19" | 5.6 months after diagnosis of COVID-19  (range 1- 12 months) | NR | Cases: CYP between the ages of 0-18 who tested positive for COVID-19 or those with a history of COVID-19 seen at the hospital.  Control: CYP between 1-18 who did not have Covid-19 and any diseases that might cause cardiac pathologies |
| Donnachie | Germany | Cases 43,903      Controls 73,873 | Cohort | Range [0-17] | NR | NR | RT-PCR (100%) | 24 months after diagnosis of COVID-19 | NR | Claims data of all patients with a physician consultation related to COVID-19, together with a control group of 1 million patients without treatment related to COVID-19 |
| Fink | Brazil | 53 cases    52 case controls | Cohort | 14.65 (8-18) | 42% | 77% mild; 11%moderate; 6% severe; 6% critical | RT-PCR) or antibody testing | 4.4 months (0.8-10.7) | 89% had pediatric pre-existing chronic conditions, including:  83% Immunosuppressive conditions  45% Others pre-existing chronic conditions  26% Autoimmune conditions | Cases: (1) symptomatic inpatients and outpatients, laboratory-confirmed SARS-CoV-2 infection  (2) 8 -18 years old |
| Funk | Includes emergency department data from 8 countries (Argentina, Canada, Costa Rica, Italy, Paraguay, Singapore, Spain and USA) | 11884 cases  1701 controls | Prospective cohort | Median 3  (IQR 0-10) | 47.2% | Severe acute illness in 18.6% of hospitalised children | Nucleic acid test | 90 days after emergency department visit | 10.8% of non-hospitalised SARS-CoV-2 positive children had a chronic underlying condition; 13.5% had a history of asthma. 25.7% of hospitalised SARS-CoV-2 positive had a chronic underlying condition; 14.8% had a history of asthma. | Children < 18 who underwent testing for SARS-CoV-2 at 39 participating pediatric emergency departments between March 7, 2020 and January 20, 2021, and completed subsequent 90-day follow-up |
| Guido | Italy | 322 | Cohort | 10 [Range 1.5-17] | 51.2% | NR | NR | 1 month and 3-5 months after infection^a^ | NR | CYP aged 1-17 years who had contracted SARS-CoV-2 referred to the post-COVID outpatient clinic of a maternal-infant ward between February and November 2021 and had parental consent to attend follow-ups |
| Haddad | Germany | 544 (140 adolescents, 404 children < 14 years old)  Infected: 334  Exposed: 210 | Part of a prospective observational cohort study | 16 (1) for adolescents,  8 (4) for children | 49.8% | NR | positive RT-  PCR or seropositive on at least 2/3 commercial antibody tests | 11-12 months | NR | Families recruited via local health authorities and an in-hospital database of households with at least one laboratory-confirmed SARS-CoV-2 infection. |
| Heiss | Germany | 54 (29 recovered and 25 long COVID cases)    9 healthy controls | Cross sectional,  prospective clinical trial | Recovered: 11±3    Long COVID: 12±3    Control: 10±3 | Recovered: 45%    Long COVID: 44%    Control: 22% | 4 participants with positive RT-PCR test were asymptomatic | RT-PCR (100%) | 222±134 days after positive RT-PCR test | 5% of recovered participants had pre-existing conditions    10% of long COVID participants had pre-existing conditions    22% of controls had pre-existing conditions | CYP aged 5- 18 years old who tested positive for SARS-CoV-2 and healthy control aged 5 to below 18 who had not tested positive for SARS-CoV-2. |
| Kikkenborg Berg, 2022a & b | Denmark | CYP:  10997  Cases    33016 Controls  Adolescents:  6630  Cases    21640 Controls | Cross Sectional | CYP  Cases:  10.2 (6.6-12.8)    Control:  10.6 (6.9-12.9)  Adolescents:  17.6 (16.4-18.5) | CYP  48%  Adolescents:58% | CYP:  54% asymptomatic  44% mild,  2% severe  Adolescents:  34% asymptomatic  57% mild, 9% severe | RT-PCR (100%) | >8 weeks after diagnosis of COVID-19* | CYP:  Cases:  0-3 years: 26% comorbidities  4-11 years:  31% had comorbidities  12-14 years: 41% comorbidities    Control:  0-3 years: 27% comorbidities  4-11 years: 40% comorbidities  12-14 years: 50% comorbidities  Adolescents:  Case: 48% had comorbidities    Control: 60% had comorbidities | CYP aged 0–14 years with a positive SARS-CoV-2 test with a matched control group who never had a positive PCR test for SARS- CoV-2 in the period between Jan 1, 2020, and July 12, 2021  Adolescents aged 15–18 years who tested positive for SARS-CoV-2 and matched controls who had not tested positive for SARS-CoV-2 in the period Jan 1, 2020, to July 12, 2021 |
| Kuczborska | Poland | Immunocompromised 70    Immunocompetent  77 | Cohort | Cases: 7.0 (4.0- 13.0)  Range [8 months- 17 years]    Control:  9.0 (4.0-13.0)  Range [4 months-17 years] | 47%        47% | Cases: 46% asymptomatic39% mild,      Control: 48% mild, 31% moderate  1% MIS-C | RT-PCR (100%) | ≥ 3 months | Allergic disease in control group 29% | Immunocompetent and immunodeficient CYP, who were admitted to the COVID-19 Subunit of a tertiary referral hospital in Warsaw, Poland. |
| Ludvigsson | Sweden | 5 | Case report | 12  Range [9-15] | 80% | 100% mild disease | CD (100%) | 6-8 months after clinical diagnosis of COVID-19 | 1 comorbidity (asthma, allergies and mild autism spectrum disorder) | Inclusion of CYP whose parents contacted the study author after experiencing symptoms more than 2 months after clinical diagnosis of COVID-19 |
| Matteudi | France | 137 | Cohort | Range 0-15 | NR | 28% asymptomatic 72% symptomatic  14% hospitalised | RT-PCR (100%) | 10-13 months after diagnosis | NR | CYP aged <16 years old that were tested positive for SARS-CoV-2 in Marseille public hospitals |
| McGrath | USA | 1080 | Retrospective cohort | 11.6±4.4 | 51.6% | 4.4% inpatient; 66.6% outpatient  3.3% emergency department | ICD-10-CM code for post-COVID-19 condition after 3 months of continuous enrollment | 3 months | 5.1% ≥1 concurrent conditions | Patients aged 0-17 with an ICD-10-CM code for post-COVID-19 condition between October 1, 2021 and January 31, 2022 |
| Messiah | USA | 1828 (58 at >12 weeks; 56 seropositive and 2seronegative) | Cross sectional | Range 5-19 years | 52.1% pre Delta variant and 50.3% in Delta variant and beyond | 89.5% mild-moderate and 4% severe in positive group | Self-reported antigen/PCR test | > 12 weeks | NR | CYP ≤ 19 years with a self-reported positive antigen/PCR COVID-19 test who completed the Texas Coronavirus Antibody REsponse Survey between October 2020 and May 2022 |
| Mohiuddin Chowdhury | Bangladesh | 3 children (age 0-10) | Cross-sectional | Range [0-10] | NR | NR | RT-PCR (100%) | ≥20 weeks after diagnosis of COVID-19 | NR | SARS-CoV-2 confirmed cases by RT-PCR identified and collected from the local health facilities of the Chattogram division in Bangladesh |
| Morrow | USA | 9 | Case series | Range [4-18] | 66.7% | NR | Nucleic acid test [NAT], serum antibody test or an acute clinical presentation consistent with COVID-19 | Range between 2-12 months^a^ | Patient 1: Type 1 diabetes, anxiety, depression  Patient 3: Speech delay  Patient 4: Anxiety, asthma, seasonal allergies  Patient 5: Dyslexia, seasonal allergies  Patient 7: Eosinophilic esophagitis, speech delay (resolved)  Patient 8: Sensory processing disorder, vitiligo, fructose malabsorption, scoliosis  Patient 9: Migraine, multiple concussions, sensory integration disorder | The first 9 patients who  presented to Pediatric Post COVID-19  Rehabilitation Clinic; < 21 years-old, self-referred with either a history of a positive COVID-19 test or an acute clinical presentation  consistent with COVID-19. |
| Ng | USA | 18 | Retrospective clinical case series | 13.28±2.70  Range [6-16] | 66.7% | Majority mild    3 required brief hospitalisation, none required mechanical ventilation | 66.7% diagnosed with nucleic acid test or serum antibody test    33.3% had an acute clinical presentation consistent with COVID-19 combined with known exposure to the illness | 280±178 days  Range [87-674] | 50% had attention/concentration problems. 72.2% had a history of anxiety or depressive symptoms. 38.8% reported both attention and mood/anxiety concerns. Four patients reported prior developmental concerns. Two presented with early motor or speech delays. Two patients had a prior diagnosis of ADHD. Five patients had a premorbid medical history significant for preterm birth ranging from 29 to 36 weeks' gestation. One patient had a history of seizure disorder | First 18 patients referred for neuropsychological evaluation from a multidisciplinary pediatric post-COVID clinic |
| Osmanov | Russia | 518 | Cohort | 10.4 (3–15.2) | 52% | All hospitalised,  3% severe | RT-PCR  (100%) | 256 days (223-271) after hospital admission | 27% had 1 comorbidity, 17% had ≥2 comorbidities | CYP ≤18 years old with RT-PCR confirmed SARS-CoV-2 infection admitted to single hospital between April and August 2020 |
| Öztürk | Turkey | 50 | Cohort | 15.3 years (8.1- 18) | 44% | All were hospitalised for acute infection. 40 had non-severe disease and 10 had severe disease (WHO classification). Nore had ARDS, sepsis, septic shock or any requirement for mechanical ventilation or vasopressor therapy. | Confirmed SARS-CoV-2 infection | 3 months |  | Patients aged 5–18 years who were hospitalized with a confirmed SARS-CoV-2 infection between May 15 and August 1, 2020, and followed up at the clinic |
| Pazukhina | Russia | 360 | Prospective cohort | Median 9.5 years  IQR (2.4-14.8) | 52% | 3% severe COVID-19 requiring non-invasive ventilation, invasive ventilation or treatment in the ICU during hospitalisation. | RT-PCR (100%) | 12±2-month follow-up | Common comorbidities included allergic rhinitis (7%) and intestinal problems (7%) | Children with positive RT-PCR confirmed SARS-CoV-2 infection hospitalised in Moscow between April and August 2020 |
| Petersen | Faroe Islands | 21 | Cohort | Range [0-17] | NR | None hospitalised | RT-PCR  (100%) | 125± 17 days  Range [45-153] after symptom onset | NR | All consecutive RT-PCR positive patients in the Faroe Islands from March to April 2020 |
| Radtke | Switzerland | Seropositive 109    Seronegative 1246 | Cohort | Range [6-16] | 53% seropositive  54% seronegative | None hospitalised | Serology (100%) | >4 weeks,  >12 weeks and  6-month follow-up after serological testing ^a^ | 16% had 1 comorbidity in seropositive group    20% had 1 comorbidity in seronegative group | Children with positive and negative serology results selected from 55 randomly selected primary and secondary schools in Zurich in October/November 2020 |
| Rao | USA | 59893 + viral test    599393 – viral test | Retrospective cohort | + viral test: 9.4±5.9    - viral test: 7.9±5.7 | + viral test: 48.7%    - viral test: 47% | 2.2% admitted to ICU | Antigen or RT-PCR | + viral test: 4.6±0.7 months    - viral test: 4.7±0.7 months | 15.8% had a non-complex chronic condition and 14.1% had a complex chronic condition. | CYP < 21 years with positive SARS-CoV-2 viral test between 1^st^ March 2020, and 31^st^ October 2021 |
| Roessler | Germany | Cases 11950    Control 59750 | Cohort | Range [0-17] | 48.1%  Cases | 98.6% outpatient, 1% hospitalised, 0.4% in ICU | 100% Laboratory confirmed diagnosis of COVID-19 | ≥3 months after COVID-19 diagnosis | 9.9% of cases had comorbidities | CYP with  documented  COVID-19  diagnosis and confirmed laboratory virus detection and CYP without COVID‐19 diagnosis  regardless of  laboratory virus  detection through June 30^th^, 2020 |
| Sante | Italy | 12  Long-COVID    17 Recovered | Cross-sectional | Long-COVID:  10.3±4.5    Recovered:  7.7±5.5 | 33% Long-COVID    36% Recovered | Long-COVID: 8% asymptomatic 92% mild, 0% hospitalised  Recovered:  12% asymptomatic, 59% mild, 18% moderate, 12% severe 29% hospitalised | RT-PCR  (100%) | 98.5 ± 41.5 “days after acute SARS-CoV-2 infection” | Long-COVID: 25% had comorbidities    Recovered:  18% had comorbidities | CYP “fully recovered or with PASC assessed in a dedicated post-  COVID outpatient service” |
| Savino | Italy | 5 | Case series | 2-15 years old | 44% | NR | RT-PCR | 3-15 months | NR | All patients < 18 years with a previous history of COVID-19 consecutively admitted to unit due to new-onset neuropsychiatric symptoms. |
| Say | Australia | 12 | Cohort | 3.7±3.5 | 42% | 92% mild,  8% severe  50% admitted to hospital (17% for observation,  8% for fluid rehydration, 25% ICU) | “Children who tested positive for SARS-CoV-2” | Range [3-6 months] after diagnosis | 17% chronic respiratory condition  8% congenital cardiac disease | CYP aged ≤18 years referred to a dedicated COVID-follow up clinic |
| Smane | Latvia | 30 | Cohort | 9.2±5.2  Range [3 months-17 years] | 43% | 17% asymptomatic  80% mild, 3% moderate,  17% hospitalised | RT-PCR (100%) | 101 ± 7 days after infection | 23% had comorbidities | SARS-CoV-2 positive CYP 0-17 years enrolled after recovery from COVID-19 at a post-acute outpatient centre |
| Stephenson | England | 3065  RT-PCR +    3739  RT-PCR - | Cohort  (Preprint) | Age: 11-15  PCR + (56%)  Age: 16-17  PCR + (44%)    Age: 11-15  PCR - (57%)  Age: 16-17  PCR - (43%) | 64% PCR +    63% PCR - | 65% of PCR + asymptomatic  35% of PCR + symptomatic    92% of PCR - asymptomatic  8% of PCR- symptomatic | RT-PCR  (100%) | 14.9 weeks (13.1-18.9) after testing | NR | SARS-CoV-2 PCR-positive CYP aged 11-17 years selected from a national database of test results held by Public Health England from January-March 2021 |
| Sterky | Sweden | 55 | Cohort | Range [<1-18] | 42% | 9 children had MIS-C, 2 of which required ICU  Other reasons for admission: 38% dehydration, 35% “infection observation”, 23% for “inhalations” | RT-PCR  (100%) | 219 days (123-324) after hospital admission | 35% had comorbidities | CYP aged 0-18 years who were admitted to one of the two paediatric hospitals in the Stockholm Region and RT-PCR positive for SARS-CoV-2 |
| Tarantino | Italy | 31 | Cross sectional | 14.1±2  Range [12-18] | 74.2% | 32% asymptomatic  68% affected by mild symptoms    One participant hospitalised and required oxygen therapy | NR | 3-6 months after infection | Patients with the presence of any pre-existing comorbidity were excluded | CYP aged 12-18 3-6 months after COVID-19 infection, without the presence of any medical conditions or other neurological disease, referred for a consultation to a children’s hospital between July 2021 to December 2021 |
| Taquet | Electronic health records from 8 countries (USA, Australia, UK, Spain, Bulgaria, India, Malaysia and Taiwan) | 185748 | Retrospective cohort study | 8.9±5.6 | 49.4% | NR | Recorded diagnosis of COVID-19 or positive test for SARS-CoV-2 | 213±204 | 7.1% overweight and obese; 2.1% hypertensive disease; 1.6% Type 2 diabetes; 14.5% asthma; 2.8% Bronchitis; 0.2% other chronic obstructive pulmonary disease; 9.4% anxiety disorders; 1.7% substance misuse; 4.8% mood disorders; 0.4% Ischemic heart disease; 4.2% heart disease; 0.6% chronic kidney disease; 4.9% neoplasms (benign or malignant) | CYP < 18 who had a confirmed diagnosis of COVID-19 recorded on the TriNetX electronic health records network between January 20, 2020 and April, 13, 2022 |
| Trapani | Italy | 629 primary care    60 hospitalised | Cohort | Primary care:  Median 8 years  Range [4-11]    Hospitalised:  Median 4 years  Range [0-11.5] | 49.8% primary care    43.3% hospitalised | 24.3% of children in primary care were symptomatic    58.3% of hospitalised children were symptomatic | Molecular swab (100%) | 2-6 months | 9.4% of children in primary care had pre-existing disease    23% of hospitalised children had pre-existing disease | CYP presenting to 18 pediatric clinics diagnosed with COVID-19 by a positive molecular swab from October 2020 to June 2021 and recovered with a negative molecular swab from at least 8 weeks |
| Werner | Germany | 45 | Cohort | Median 10 years  Range [0-18] | 64% | 89% asymptomatic or mild, 11% moderate | NR (Patients who fulfilled the long COVID diagnostic criteria) | 14.3 weeks  Range [7 weeks – 9 months] | 15 patients had a current or previous medical issue; 6 patients had more than one. 10 patients had a pulmonary/allergic disease, with 7 having a history of atopy. Two patients had cystic fibrosis. Two patients had obesity    Two patients had a current seizure disorder. One patient had a diagnosis of metabolic syndrome and Rolandic epilepsy. One patient had absence seizures  . One patient had autoimmune hepatitis/primary sclerosing hepatitis overlap. No patients had a diagnosis of obstructive sleep apnea | CYP who presented with sleeping disorders to the interdisciplinary long COVID outpatient clinic a children’s hospital for the first time, from January 2021 to May 2022 |

*data extracted for the longest follow up point (>12 weeks after infection)
