# Supplementary material for: Persistent symptoms are associated with long term effects of COVID-19 among children and young people: Results from a systematic review and meta-analysis of controlled studies
Source: PLoS One. 2023 Dec 28;18(12):e0293600. doi: 10.1371/journal.pone.0293600 (PMC10754445; doi:10.1371/journal.pone.0293600)
Supplement: S3 Table — (DOCX) [file pone.0293600.s005.docx]

# **S3 Table- Symptoms reported in <3 controlled studies and therefore not included in the meta-analysis**

| Menstruation changes |
| --- |
| Chills |
| Hoarse voice |
| Earache / ringing in ears |
| Discoloured fingers and toes |
| Cold hands and feet |
| Mood swings |
| “Neurological symptoms” |
| Seizures / fits |
| Light sensitivity |
| Eye soreness |
| Cracked red lips |
| Problems speaking communicating |
| Urination problem |
| Weight loss |
| Joint pain / swelling |
| Hair loss |
| “Psychiatric symptoms” |
| Pulmonary Embolism |
| Decreased physical resilience |
| Less motivation |
| Nervousness |
| Weight gain |
