# Supplementary material for: Persistent symptoms are associated with long term effects of COVID-19 among children and young people: Results from a systematic review and meta-analysis of controlled studies
Source: PLoS One. 2023 Dec 28;18(12):e0293600. doi: 10.1371/journal.pone.0293600 (PMC10754445; doi:10.1371/journal.pone.0293600)
Supplement: S4 Table — (DOCX) [file pone.0293600.s006.docx]

# **S4 Table- Pooled prevalence estimates for symptoms reported by CYP with PCC**

| **Symptom** | **Number of studies**  **(N)** | **Number of CYP with symptom**  **(N)** | **Total CYP with LC**  **(N)** | **% prevalence** | **Lower CI** | **Upper CI** |
| --- | --- | --- | --- | --- | --- | --- |
| Anxiety | 8 | 2173 | 12556 | 17.3% | 16.6% | 18.0% |
| Chest pain/tightness | 14 | 2498 | 13369 | 18.7% | 18.0% | 19.4% |
| Cognitive difficulties | 17 | 453 | 2053 | 22.1% | 20.3% | 23.9% |
| Cough | 18 | 4812 | 13869 | 34.7% | 33.9% | 35.5% |
| Depression | 6 | 1488 | 12186 | 12.2% | 11.6% | 12.8% |
| Dermatological symptoms | 5 | 134 | 671 | 20.0% | 16.9% | 23.0% |
| Dizziness | 12 | 270 | 1550 | 17.4% | 15.5% | 19.3% |
| Dyspnoea | 14 | 167 | 1329 | 12.6% | 10.8% | 14.4% |
| Fatigue/weakness | 25 | 2315 | 14187 | 16.3% | 15.7% | 16.9% |
| Fever | 13 | 3515 | 13620 | 25.8% | 25.1% | 26.5% |
| Headache | 23 | 5089 | 14291 | 35.6% | 34.8% | 36.4% |
| Cardiovascular symptoms | 12 | 240 | 1801 | 13.3% | 11.8% | 14.9% |
| Loss of appetite | 8 | 76 | 1147 | 6.6% | 5.2% | 8.1% |
| Loss or altered smell or taste | 15 | 94 | 1176 | 8.0% | 6.4% | 9.5% |
| Myalgia | 14 | 398 | 1776 | 22.4% | 20.5% | 24.3% |
| Nasal congestion | 3 | 38 | 292 | 13.0% | 9.2% | 16.9% |
| Ophthalmologic and/or otolaryngologic symptoms | 5 | 2025 | 12118 | 16.7% | 16.0% | 17.4% |
| Rash | 11 | 297 | 1704 | 17.4% | 15.6% | 19.2% |
| Sleep difficulty | 10 | 138 | 813 | 17.0% | 14.4% | 19.6% |
| Sore Throat | 6 | 2598 | 13018 | 20.0% | 19.3% | 20.6% |
| Gastrointestinal problems | 15 | 6909 | 13769 | 50.2% | 49.3% | 51.0% |
