# Supplementary material for: Persistent symptoms are associated with long term effects of COVID-19 among children and young people: Results from a systematic review and meta-analysis of controlled studies
Source: PLoS One. 2023 Dec 28;18(12):e0293600. doi: 10.1371/journal.pone.0293600 (PMC10754445; doi:10.1371/journal.pone.0293600)
Supplement: S5 Table — (DOCX) [file pone.0293600.s007.docx]

# **Table 5- Pooled prevalence estimates for symptoms reported by SARS-CoV-2 infected CYP**

| **Symptom** | **Number of studies** | **Number of CYP with symptom** | **Total CYP with SARS-CoV-2 infection** | **% prevalence** | **Lower CI** | **Upper CI** |
| --- | --- | --- | --- | --- | --- | --- |
| Anxiety | 8 | 227 | 8376 | 2.7% | 2.4% | 3.1% |
| Chest pain/tightness | 11 | 283 | 6914 | 4.1% | 3.6% | 4.6% |
| Cognitive difficulties | 21 | 690 | 58357 | 1.2% | 1.1% | 1.3% |
| Cough | 18 | 403 | 9618 | 4.2% | 3.8% | 4.6% |
| Depression | 5 | 72 | 2891 | 2.5% | 1.9% | 3.1% |
| Dermatological symptoms | 8 | 96 | 5240 | 1.8% | 1.5% | 2.2% |
| Dizziness | 8 | 444 | 6715 | 6.6% | 6.0% | 7.2% |
| Dyspnoea | 16 | 2484 | 50449 | 4.9% | 4.7% | 5.1% |
| Fatigue | 28 | 5065 | 61345 | 8.3% | 8.0% | 8.5% |
| Fever | 12 | 134 | 7930 | 1.7% | 1.4% | 2.0% |
| Headache | 23 | 1174 | 16048 | 7.3% | 6.9% | 7.7% |
| Cardiovascular symptoms | 12 | 116 | 5191 | 2.2% | 1.8% | 2.6% |
| Loss of appetite | 7 | 355 | 58659 | 5.4% | 5.2% | 5.6% |
| Loss or altered smell or taste | 22 | 1442 | 58659 | 2.5% | 2.3% | 2.6% |
| Myalgia | 13 | 1011 | 52560 | 1.9% | 1.8% | 2.0% |
| Nasal congestion | 5 | 55 | 4116 | 1.3% | 1.0% | 1.7% |
| Ophthalmologic and/or otolaryngologic symptoms | 2 | 26 | 2137 | 1.2% | 0.8% | 1.7% |
| Rash | 7 | 48 | 3261 | 1.5% | 1.1% | 1.9% |
| Sleep difficulty | 15 | 259 | 8184 | 3.2% | 2.8% | 3.5% |
| Sore Throat | 3 | 295 | 3688 | 8.0% | 7.1% | 8.9% |
| Gastrointestinal problems | 16 | 390 | 8573 | 4.6% | 4.1% | 5.0% |
